# FGF401 and vinorelbine synergistically mediate antitumor activity and vascular normalization in FGF19-dependent hepatocellular carcinoma

**DOI:** 10.1038/s12276-020-00524-4

**Published:** 2020-11-25

**Authors:** Hung Huynh, Aldo Prawira, Thi Bich Uyen Le, Thanh Chung Vu, Huai-Xiang Hao, Alan Huang, Youzhen Wang, Diana Graus Porta

**Affiliations:** 1 Laboratory of Molecular Endocrinology, Division of Molecular and Cellular Research, National Cancer Centre, Singapore, Singapore; 2 Oncology Drug Discovery Pharmacology, Novartis Institutes for Biomedical Research, 250 Massachusetts Avenue, Cambridge, MA 02139 USA; 3 Oncology Translational Research, Novartis Institutes for Biomedical Research at Basel, Basel, Switzerland

**Keywords:** Cancer models, Targeted therapies

## Abstract

Hepatocellular carcinoma (HCC) is a lethal cancer with limited therapeutic options, and standard therapy with sorafenib provides only modest survival benefits. Fibroblast growth factor 19 (FGF19) has been proposed as a driver oncogene, and targeting its receptor, FGFR-4, may provide a better alternative to standard therapy for patients with FGF19-driven tumors. Sixty-three HCC patient-derived xenograft (PDX) models were screened for FGF19 expression. Mice bearing high and low FGF19-expressing tumors were treated with FGF401 and/or vinorelbine, and the antitumor activity of both agents was assessed individually and in combination. Tumor vasculature and intratumoral hypoxia were also examined. High FGF19 expression was detected in 14.3% (9 of 63) of the HCC models tested and may represent a good target for HCC treatment. FGF401 potently inhibited the growth of high FGF19-expressing HCC models regardless of *FGF19* gene amplification. Furthermore, FGF401 inhibited the FGF19/FGFR-4 signaling pathway, cell proliferation, and hypoxia, induced apoptosis and blood vessel normalization and prolonged the overall survival (OS) of mice bearing high FGF19 tumors. FGF401 synergistically acted with the microtubule-depolymerizing drug vinorelbine to further suppress tumor growth, promote apoptosis, and prolong the OS of mice bearing high FGF19 tumors, with no evidence of increased toxicity. Our study suggests that a subset of patients with high FGF19-expressing HCC tumors could benefit from FGF401 or FGF401/vinorelbine treatment. A high level of FGF19 in a tumor may serve as a potential biomarker for patient selection.

## Liver cancer: combining two drugs for improved therapeutic effect

The drugs FGF401 and vinorelbine, when working together synergistically, could be effective in treating those liver cancers driven by the activity of the fibroblast growth factor 19 (FGF19) protein. The drugs’ effects on human tumors grafted into mice were studied by an international research team led by Hung Huynh at the National Cancer Centre in Singapore. FGF401 is a small molecule that inhibits the activity of the receptor protein that the FGF19 growth factor interacts with to promote some cancers. Vinorelbine disrupts protein microtubules required for the cell division that allows cancer cells to multiply. In combination, the drugs achieved significantly enhanced anti-cancer effects which can now be tested in clinical trials. The research also uncovered new details of FGF401’s therapeutic actions, including its ability to restore healthy blood vessel formation.

## Introduction

The diagnosis of hepatocellular carcinoma (HCC) portends a dismal diagnosis and is not amenable to existing therapeutic modalities^1^. Two multikinase inhibitors, sorafenib^2,3^ and lenvatinib^4^, are currently approved by the FDA as the first line of treatment for advanced HCC. Although sorafenib^2,3^ and lenvatinib^4^ improve the overall survival (OS) of patients with HCC, the benefit is modest at best and transient clinically. A randomized phase III study evaluating nivolumab versus sorafenib as a first-line treatment in HCC patients (NCT02576509) did not meet its primary endpoint of OS. Clearly, there is a need for effective novel therapies to combat this deadly disease.

Recent data have indicated fibroblast growth factor (FGF) 19 (FGF19) as one of the driver oncogenes in HCC^5,6^. FGF19 has high specificity for its receptor FGFR-4, and its binding to the FGFR-4/Klotho-β complex results in the activation of the Ras-Raf-Erk1/2 and PI3K-Akt pathways, which are involved in cell proliferation and antiapoptosis^7,8^. FGF19 gene amplification and FGF19 overexpression are detected in approximately 14 and 50% of HCC patients, respectively^9,10^. Moreover, FGF19 expression correlates with a poor prognosis, recurrence, tumor progression, and short OS^5,10^. Overexpression of FGF19 in mice results in liver cancers that are sensitive to anti-FGFR-4 and anti-FGF19 antibodies^9,11,12^.

FGFR-4 expression is higher in HCC tissues than in adjacent normal liver tissues^13^ and is associated with a later TNM stage and shorter OS^14^. Overexpression of FGFR-4 in vitro leads to increased invasion, while its downregulation results in decreased viability, invasion, and tumor formation^5,15^. Knockout of FGFR-4 prevented transgenic mice that exogenously expressed FGF19 from developing tumors, suggesting an association of aberrant FGF19/FGFR-4 in the development of HCC and providing a rationale for targeting FGF19/FGFR-4 to treat a subgroup of patients with FGF19/FGFR-4-dependent HCC tumors^9,12,16,17^. Indeed, evidence of antitumor activity was seen in a phase I study of fisogatinib in patients with FGF19-positive HCC^18^.

NVP-FGF401 (FGF401) is a highly potent and selective FGFR-4 inhibitor (IC50 = 3 nmol/L) but shows weak inhibitory activity against FGFR-1 (IC50 = 591 nmol/L), FGFR-2 (IC50 = 493 nmol/L), and FGFR-3 (IC50 = 150 nmol/L). In vivo, FGF401 exposure near the IC90 dose is required to achieve tumor regression^19^. Moreover, FGF401 inhibits tumor xenografts that express FGF19, FGFR-4, and Klotho-β^19^. An ongoing phase I/II study of FGF401 in patients with HCC or solid tumors with positive FGFR-4 and Klotho-β expression (NCT02325739) suggested promising clinical activity and a manageable safety profile that is consistent with FGFR-4 pathway inhibition. Studies to further evaluate FGF401 as a single agent and in combination are ongoing^20^.

In the present study, we aimed to better understand the mechanisms that underlie the growth inhibition of FGF401. First, we investigated whether FGF401 affects tumor angiogenesis, tumor hypoxia, and the overall survival (OS) of mice bearing orthotopic HCC. Second, we determined whether the slowing of tumor growth resulted from reduced tumor cell proliferation, increased tumor cell death, or both. Finally, we determined whether the addition of vinorelbine improved the antitumor activity of FGF401. Here, we report the effects of FGF401 monotherapy and its combination with vinorelbine in high FGF19-expressing models of human HCC.

## Materials and methods

The reagents, isolated HCC cells and cultures, copy number studies, Western blot analyses, immunohistochemistry analyses, cell cycle analyses, vessel perfusion studies, and statistical analyses were performed as previously described^21^. Detailed methods are available in the Supplementary Materials and Methods.

For the FGF19-stimulated activation of FGFR, freshly isolated HCC cells were treated with vehicle or 1 µM FGF401 for 24 h and then stimulated with 200 ng/ml recombinant FGF19 for 30 min. Cells were harvested, and changes in the levels of proteins were determined by Western blot analysis^21^.

### Efficacy of FGF401 in ectopic HCC models

For the dose-response experiment, mice bearing HCC25-0705A xenografts (*n* = 10 per group) were orally given 5% dextrose (vehicle) or FGF401 (20, 30, and 40 mg/kg) twice daily (BID) for the indicated days. For the time-dependent inhibition of FGF401 targets, mice bearing HCC25-0705A tumors were orally administered 200 µl of either vehicle (*n* = 6) or 60 mg/kg FGF401 (*n* = 10) BID. Tumors were collected at the end of the treatment time points for subsequent analyses.

To investigate the antitumor effects of FGF401, 8–10 mice per group bearing high or low FGF19-expressing tumors were orally administered vehicle or 30 mg/kg FGF401 BID for 10–28 days. For the FGF401/vinorelbine combination study, mice bearing tumors were treated as follows: (1) oral administration of 200 µl of vehicle BID (control), (2) 30 mg/kg FGF401 BID, (3) IP injection of 3 mg/kg vinorelbine every 3.5 days, and (4) combined oral FGF401 and injectable vinorelbine at the aforementioned frequency. All treatments started when the tumor sizes reached approximately 170–250 mm^3^. Body weight and signs of illness were recorded daily, and tumor volume over time was recorded until the end of the study and calculated as previously described^21,22^. Tumor tissues were harvested 2 h after the last treatments for subsequent analyses.

### Efficacy of FGF401 and FGF401/vinorelbine in orthotopic HCC models

HCC09-0913 and HCC25-0705A orthotopic models were generated as previously described^23^. For the survival study, 10 mice per group bearing tumors were treated with vehicle, vinorelbine, FGF401, or the combination of FGF401 and vinorelbine as described above for 28 days. Treatments began when the tumor sizes reached approximately 100–150 mm^3^. Body weight and overall survival were monitored daily. Tumor-bearing mice were sacrificed when they became moribund, and the presence of ascitic fluid in each mouse was recorded. The size of the primary orthotopic tumor was also documented.

## Results

### Expression of FGFR-4, Klotho-β, and FGF19 in 63 HCC patient-derived xenograft (PDX) models and the mechanism of FGF401 in vitro and in vivo

**Fig. 1:**
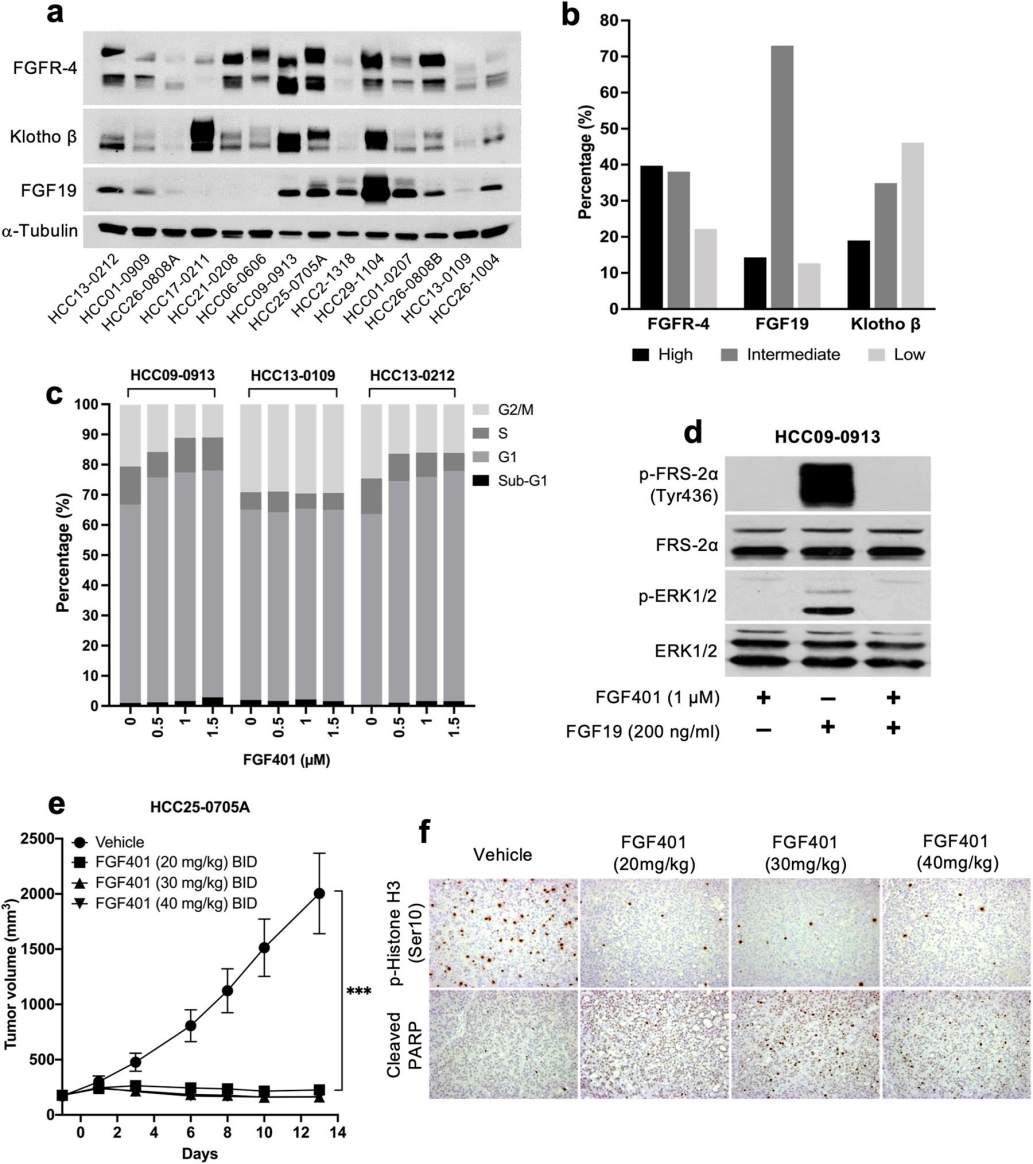
FGFR-4, FGF19, and Klotho-β expression and the effects of FGF401 in vitro and in vivo. Representative blots of FGFR-4, FGF19, and Klotho-β in 14 of 63 established HCC models are shown (**a**) and were classified as high, intermediate, or low (**b**). Cells were treated with FGF401 and subjected to cell cycle analysis (**c**). HCC09-0913 cells were pretreated with vehicle or FGF401 and then stimulated with FGF19 (**d**). Mice were orally administered FGF401 twice daily. Mean tumor volumes ± SE are plotted (*p < 0.01; Student’s *t* test) (**e**). Tumor s**e**ctions were stained for p-Histone H3 Ser10 and cleaved PARP (**f**).

High and intermediate levels of FGFR-4 were detected in 39.7% (25/63) and 38.1% (24/63) of HCC models, respectively, while high and intermediate levels of Klotho-β were observed in 19.0% (12/63) and 34.9% (22/63) of HCC PDXs, respectively. High (mRNA levels ≥ 10) and low (mRNA levels > 0.0 and < 2.0) FGF19 expression levels were found in 14.3% (9/63) and 12.7% (8/63) of HCC models, respectively (Fig. 1a, b; Supplementary Fig. 1a, b). WES analysis revealed that none of the xenograft lines tested harbored any new or known mutations in FGFR-4 or FGF19. Of the PDX models tested, 20% (3/15) and 13.3% (2/15) had 3 and 4 *FGFR-4* copy numbers, respectively. The amplification of *FGFR-4* (copy number ≥ 3) was common among HCC PDX models with high FGFR-4 expression (Supplementary Fig.1b). Among the nine high FGF19-expressing lines tested, only HCC29-1104 had FGF19 amplification.

HCC29-1104, HCC26-0808B, HCC25-0705A, HCC09-0913, HCC01-0207, HCC26-1004, HCC2-1318, HCC13-0212, and HCC10-0112B models showed high FGF19 levels among all the tested PDXs and were therefore chosen for FGF401 testing. HCC13-0109, HCC26-0808A, HCC06-0606, and HCC17-0211 models had very low to undetectable levels of FGF19 and were used as negative controls (Fig. 1a).

FGF401 increased the percentage of cells in the G1 and sub-G1 phases with a concomitant decrease in the percentage of cells in the G2/M and S phases, suggesting that FGF401 causes G1 cell cycle arrest (Fig. 1c). We then assessed the phosphorylation of FRS-2α at Tyr436 and p-Erk1/2 as a measure of FGF19/FGFR-4 signaling activity. FGF19 stimulated the phosphorylation of FRS-2α and the downstream signaling molecule Erk1/2; however, this was abolished when cells were pretreated with FGF401 for 24 h, suggesting the ability of FGF401 to inhibit the FRS-2α/Erk1/2 pathway (Fig. 1d).

Next, we determined the optimal FGF401 dose in vivo. Treatment of mice bearing high FGF19-expressing HCC25-0705A tumors with 20, 30, and 40 mg/kg FGF401 twice a day led to 83.5%, 86.8, and 87% reductions in tumor burden, respectively (*p* < 0.0001, Fig. 1e). Moreover, FGF401 significantly reduced p-Histone H3 Ser10 and increased the proportion of cleaved PARP-positive cells in a dose-dependent manner (Fig. 1f). Moreover, the suppression of p-FRS-2α and p-Erk1/2 and the increase in cleaved caspase 3 occurred within 3–12 h after drug administration (Supplementary Fig. 2a). Subsequently, 30 mg/kg FGF401 was chosen for further studies due to minimal toxicity and nearly identical efficacy as 40 mg/kg.

Importantly, no significant body weight loss or acute mortality was observed over the course of treatment, indicating minimal toxicity associated with the administration of 30 mg/kg FGF401 twice daily. Furthermore, FGF401 caused a significant elevation in lymphocytes, monocytes, and platelets and in serum ALT, ALP, and AST levels as well as a decrease in serum creatinine levels (Supplementary Table 1).

### FGF401 inhibits FGF19/FGFR-4 signaling, induces apoptosis, and normalizes blood vessels in high FGF19-expressing HCC models

**Fig. 2:**
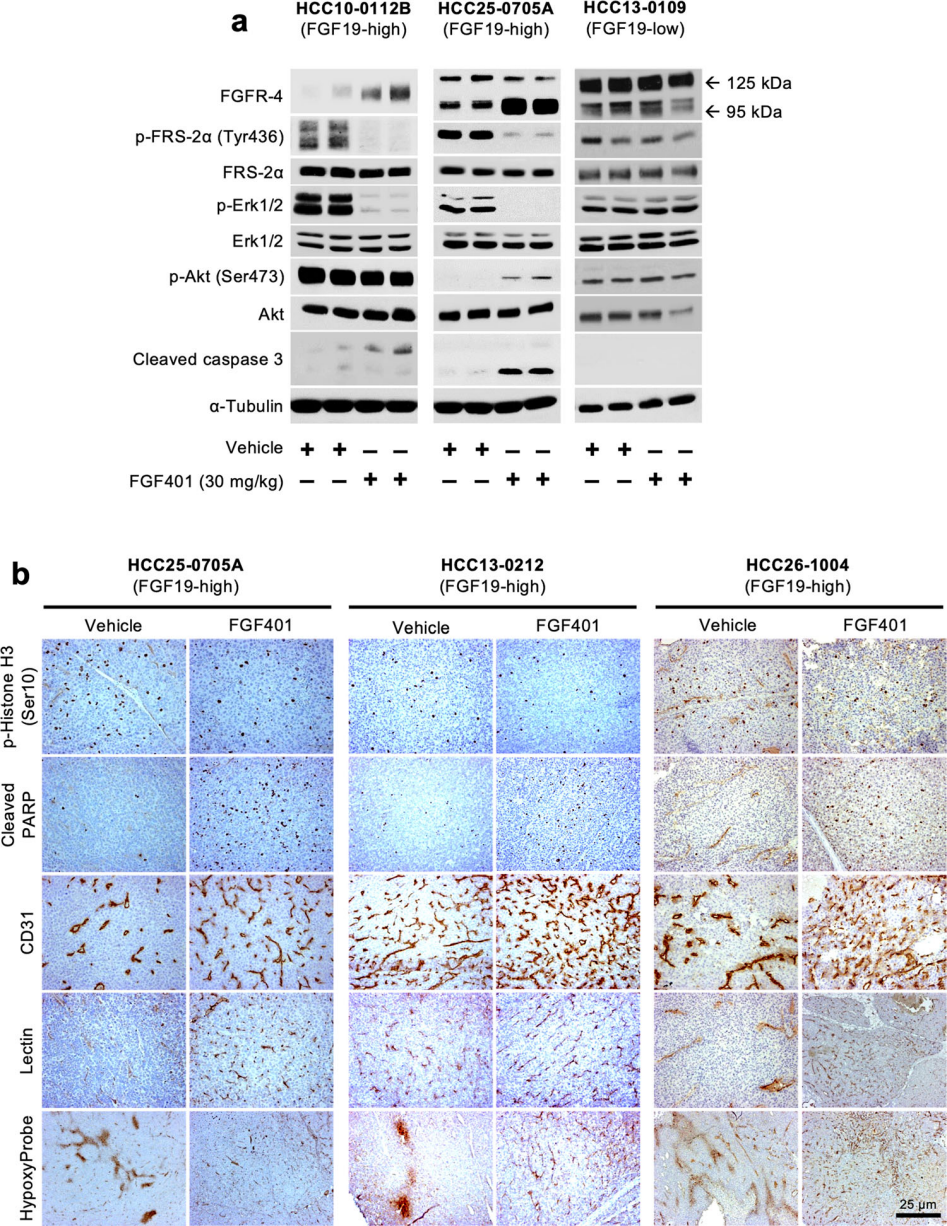
Effects of FGF401 on the FGFR-4 signaling pathway, cell apoptosis, and blood vessel normalization in high FGF19-expressing models. Lysates from 2 independent tumors treated with vehicle or FGF401 were subjected to Western blot analysis. FGF401 suppresses the FGF19/FGFR-4 signaling pathway in high FGF19-expressing tumors but not in low FGF19-expressing tumors (**a**). FGF401 increases the number of p-Histone H3 Ser10- and cleaved PARP-positive cells, increases blood vessel density, and reduces hypoxia (**b**).

The expression of FGFR-4 (95 kDa band) was higher in FGF401-treated high FGF19-expressing HCC10-0112B and HCC25-0705A tumors than in control tumors. This 95 kDa FGFR4 band migrated faster than the 125 kDa FGFR4 band in the vehicle-treated tumors, possibly due to reduced FGFR-4 phosphorylation and glycosylation. Consistent with these results, FGF401 treatment suppressed the levels of p-FRS-2α and p-Erk1/2 and increased the levels of the apoptosis marker cleaved caspase 3 in HCC10-0112B and HCC25-0705A tumors. However, this effect was not observed in low FGF19-expressing HCC13-0109 tumors (Fig. 2a).

High FGF19-expressing tumors treated with FGF401 had a significantly lower proportion of p-Histone 3 Ser10-positive cells and a higher proportion of cleaved PARP-positive cells than vehicle-treated tumors (Fig. 2b and Supplementary Fig. 2b; *p* < 0.001). No significant change in tumor cell proliferation or the percentage of cleaved PARP-positive cells was observed in low to undetectable FGF19-expressing HCC10-0505, HCC26-0808A, or HCC01-1214 tumors following FGF401 treatment (Supplementary Fig. 2b). This finding suggests that an increase in cell death and a decrease in cell proliferation were responsible in part for the observed tumor growth inhibition.

Furthermore, high FGF19-expressing tumors from mice treated with FGF401 showed significantly higher vessel counts than those from mice treated with vehicle (*p* < 0.05). Blood vessels in vehicle-treated tumors were irregularly shaped and tortuous, while blood vessels in FGF401-treated tumors remained slim, resembling capillary-like vessels (Fig. 2b and Supplementary Fig. 2b). Interestingly, FGF401 did not cause similar changes in very low and undetectable FGF19-expressing tumors (Supplementary Fig. 2b). In high FGF19-expressing HCC26-1004 tumors, treatment with the pan-FGFR inhibitor infigratinib did not induce vascular normalization, suggesting that changes in blood vessel morphology are due specifically to FGF401 inhibition of the FGF19/FGFR-4 axis (Supplementary Fig. 3). In addition, the number of lectin-positive vessels was significantly increased in FGF401-treated high FGF19-expressing tumors (Fig. 2b), suggesting that they were well perfused and productive. In contrast, blood vessels in vehicle-treated tumors were negative or weakly stained for lectin, indicating that they were nonfunctional. This was further corroborated by the absence of pimonidazole staining in FGF401-treated tumors, while vehicle-treated tumors showed interspersed hypoxic regions, implying that FGF401 restores intratumoral oxygenation (Fig. 2b). Together, the tumor hypoxia and perfusion analyses suggest that FGF401 normalizes blood vessel functions.

### FGF401 demonstrates potent antitumor activity and prolongs survival in high FGF19-expressing ectopic and orthotopic HCC models

**Fig. 3:**
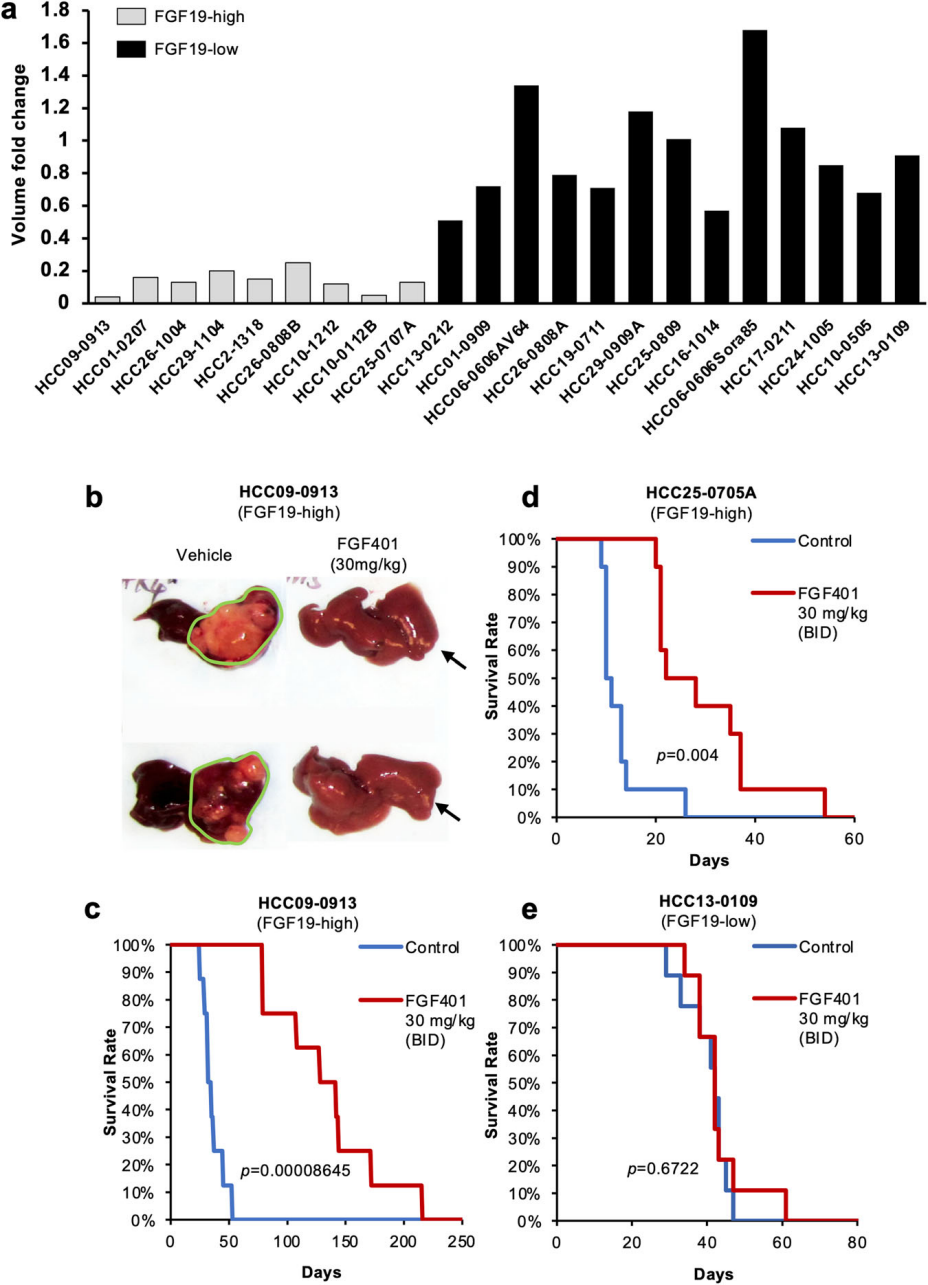
FGF401 demonstrates potent antitumor activity and prolongs mouse survival in high FGF19-expressing HCC models. The efficacy of 30 mg/kg FGF401 on high and low FGF19-expressing HCC models was determined as the volume fold change relative to the vehicle control (**a**). Orthotopic models were established, and mice were treated with vehicle or 30 mg/kg FGF401 twice daily for 28 days. The sizes of the primary tumors (**b**) and Kaplan-Meier survival analyses (**c**, **d**, and **e**) are shown (log-rank test).

We further investigated the antitumor activity of FGF401 in high and very low to undetectable FGF19-expressing models. The oral administration of 30 mg/kg FGF401 twice a day was effective against all 9 models that expressed high levels of FGF19, as indicated by the fold change in tumor volume at the end of treatment. In contrast, HCC models that expressed very low to undetectable levels of FGF19 were insensitive to FGF401 (Fig. 3a and Supplementary Fig. 4). The growth of the HCC17-0211, HCC06-0606, and HCC21-0208 models, which express high levels of FGFR-4 and Klotho-β but undetectable levels of FGF19, was not inhibited by FGF401 (Fig. 3a). These results suggest that FGF401 growth inhibition is specific to high FGF19-expressing tumors and that it targets FGF19/FGFR-4 autocrine/paracrine/intracrine loops. Similar results were obtained from other HCC lines tested (Supplementary Table 2).

In orthotopic models, vehicle-treated mice developed a swollen abdomen and became highly cachetic within 16 (HCC25-0705A) and 35 (HCC09-0913) days after tumor inoculation. Upon autopsy, large intrahepatic tumors were found, while FGF401 treatment potently inhibited tumor growth, leaving small-sized tumors in the liver (Fig. 3b; *p* < 0.001). Kaplan–Meier survival analysis revealed that all mice in the vehicle-treated group were moribund at day 52 (HCC09-0913) and day 22 (HCC25-0705A); however, the survival of the FGF401-treated mice was substantially longer (moribund at day 212 for HCC09-0913, Fig. 3c, *p* = 0.000086; and at day 56 for HCC25-0705A, Fig. 3d, *p* = 0.004). Conversely, FGF401 did not prolong the survival of mice bearing low FGF19-expressing HCC13-0109 tumors (Fig. 3e; *p* = 0.6722), indicating that FGF401 conferred a survival benefit in high FGF19 tumor-bearing mice.

### FGF401 selectively inhibits FGF19/FGFR-4 signaling

**Fig. 4:**
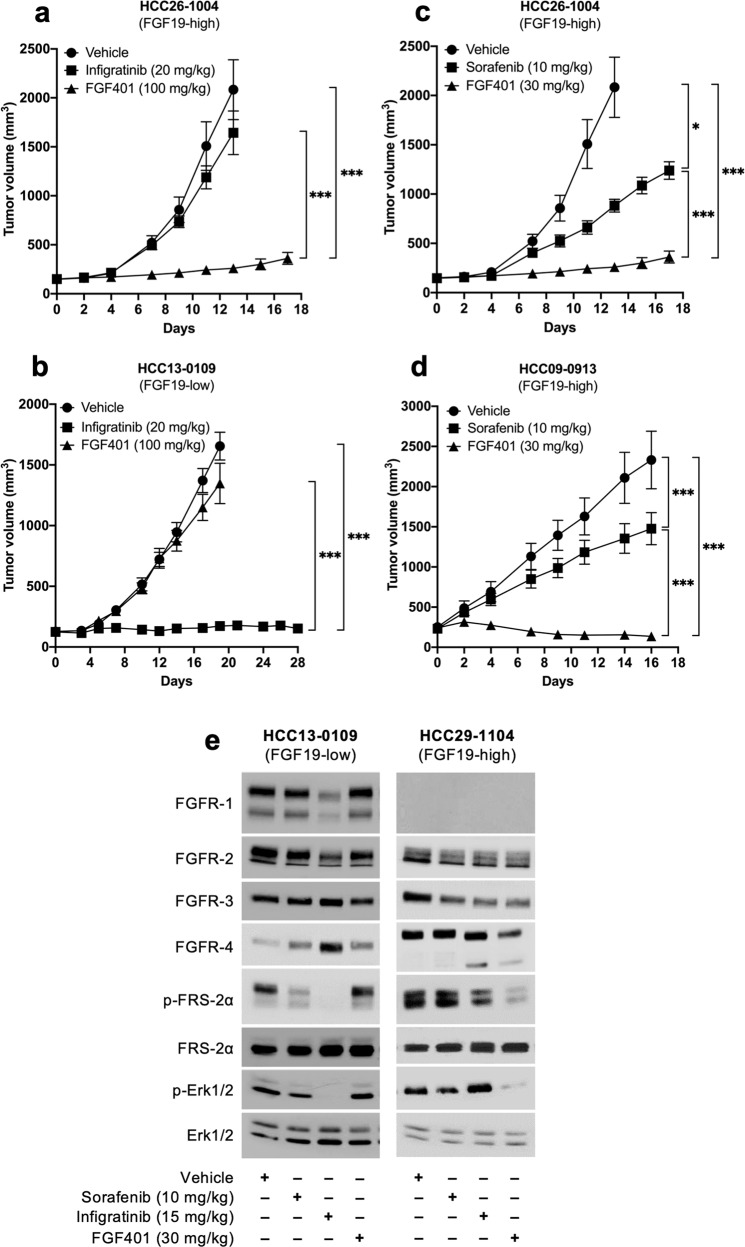
The efficacy of FGF401 vs. sorafenib and infigratinib and the specificity of FGF401 on the FGF19/FGFR-4 pathway. Mice were treated with vehicle, FGF401 twice daily or infigratinib once daily (near MTDs). Mean tumor volumes ± SEs are shown (**a**, **b**). Mice were treated with vehicle, FGF401 twice daily or sorafenib once daily (optimal doses), and the mean tumor volumes ± SEs are plotted (**c**, **d**). Mice were treated with vehicle, FGF401, infigratinib or sorafenib for 3 days, and tumors collected 2 h after the last dose were analyzed by Western blotting (**e**). *p < 0.01; **p < 0.001.

The antitumor activity of FGF401 was compared with that of infigratinib and sorafenib in high and low FGF19-expressing models. Infigratinib showed no inhibitory activity against the growth of HCC26-1004 tumors, which highly express FGF19 (Fig. 4a). However, infigratinib significantly inhibited the growth of HCC13-0109 tumors, which express FGFR-4 and Klotho-β but not FGF19 (Fig. 4b). Sorafenib inhibited tumor growth by approximately 50 and 30% in the HCC26-1004 and HCC09-0913 models, respectively. In both FGF19-high HCC26-1004 and HCC09-0913 models, FGF401 showed superior antitumor activity than sorafenib (Fig. 4c, d).

Next, we sought to demonstrate the selectivity of FGF401 in inhibiting FGF19/FGFR-4 signaling. FGF401 demonstrated a potent reduction in p-FRS-2α and p-ERK1/2 in the high FGF19-expressing HCC29-1104 model. Conversely, infigratinib demonstrated a reduction in p-FRS-2α and p-ERK1/2 in the HCC13-0109 model, which does not express detectable levels of FGF19 (Fig. 4e). This result suggests the selectivity of FGF401 for FGF19/FGFR-4 versus FGFR-1/2/3 and that the antitumor activity of FGF401 is specific to tumors with high FGF19/FGFR-4 expression. Western blot analyses of HUVECs showed that the levels of p-ERK1/2, p-Akt, p-Rb, p-p27, p27, p-p70S6K, and p-S6R were not significantly altered following 24 h of treatment with FGF401 (Supplementary Fig. 5a). Since HUVECs do not express FGF19 and FGFR-4, this further highlights the specificity of FGF401.

### FGF401 potentiates the antitumor activity of vinorelbine to inhibit tumor growth, cell proliferation and positive cell cycle regulators and prolongs the survival of mice bearing high FGF19-expressing HCC tumors

**Fig. 5:**
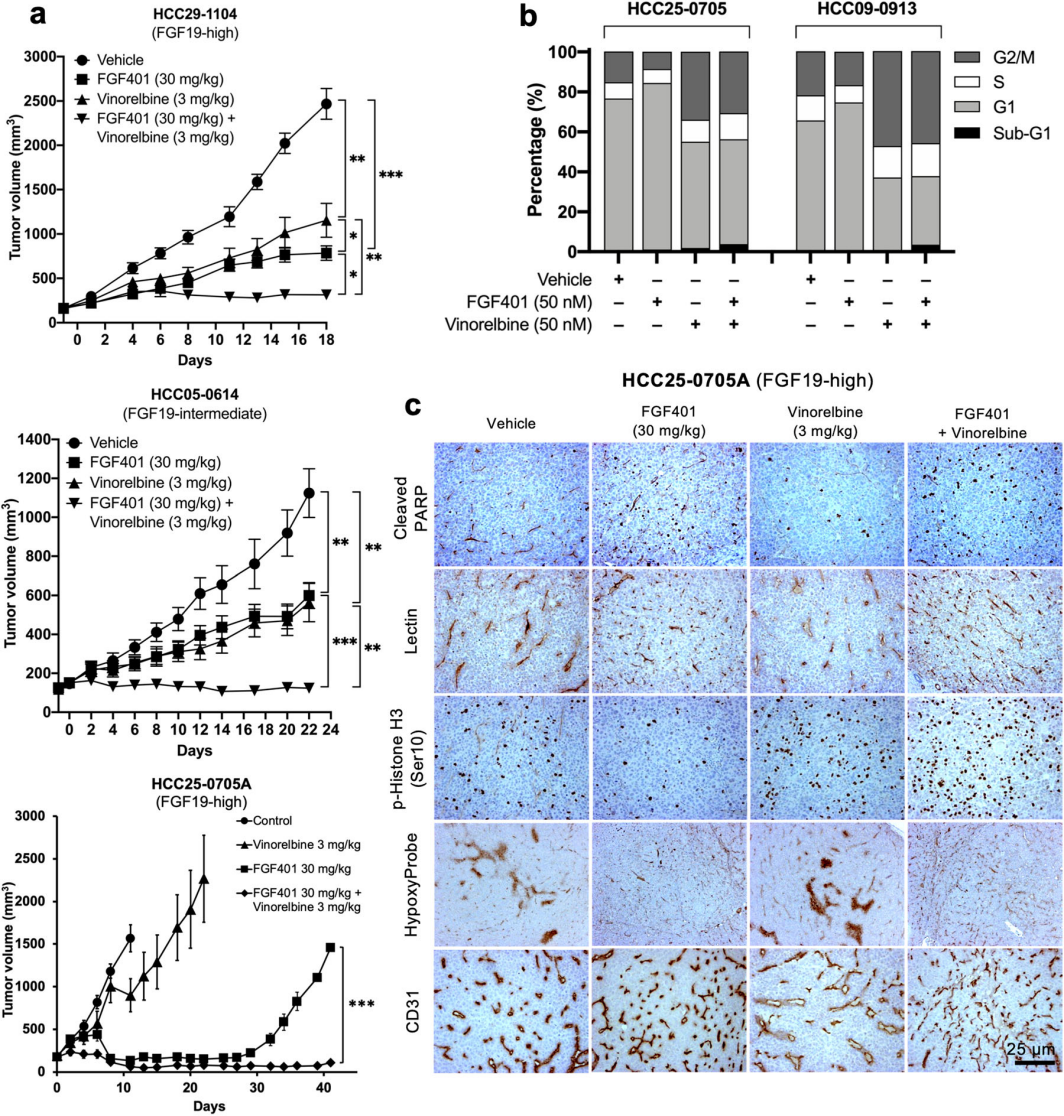
Effects of FGF401, vinorelbine and FGF401 plus vinorelbine on tumor growth, cell proliferation, apoptosis, angiogenesis, tumor hypoxia, and blood vessel normalization in high FGF19-expressing HCC models. Tumors were implanted subcutaneously, and mice were randomized into groups and treated with 200 µl of vehicle, 30 mg/kg FGF401 twice daily, 3 mg/kg vinorelbine once every 3.5 days or the combination of FGF401 plus vinorelbine. The mean tumor volumes ± SEs are plotted (**a**). Primary cultures from HCC25-0705A and HCC09-0913 tumors were treated with vehicle, FGF401 or vinorelbine for 24 h and subjected to cell cycle analysis (**b**). HCC25-0705A tumors were stained for cleaved PARP, lectin, p-Histone H3 Ser10, hypoxia (Hypoxyprobe), and CD31 (blood vessels) (**c**). *p < 0.01; **p < 0.001.

To investigate whether the antitumor activity of FGF401 is augmented by the addition of vinorelbine, mice were treated with vehicle, FGF401, vinorelbine, or FGF401 plus vinorelbine as previously described. In all high FGF19-expressing models, the addition of vinorelbine inhibited tumor growth more completely than did FGF401 and vinorelbine treatment alone (Fig. 5a). Furthermore, cell cycle analyses suggested that FGF401 and vinorelbine increased the proportion of cells in sub-G1 and induced G2/M arrest (Fig. 5b). Similarly, the addition of FGF401 further reduced the tumor burden in the other lines tested (Supplementary Table 3). These results suggest that FGF401 enhances the antitumor activity of vinorelbine.

Vinorelbine-treated HCC25-0705A tumors had a 1.6- to 1.8-fold higher proportion of p-Histone H3 Ser10-positive cells than vehicle-treated tumors, probably due to the ability of vinorelbine to arrest cells in mitosis^24^. FGF401 had no significant effect on the vinorelbine-induced elevation of p-Histone H3 Ser10-positive cells (Fig. 5c). Interestingly, the FGF401/vinorelbine combination had 1.7- to 2-fold and 5- to 6-fold more cleaved PARP-positive cells than FGF401 and vinorelbine treatment alone, respectively (Fig. 5c, *p* < 0.01).

Moreover, FGF401-treated tumors had increased productive blood vessels and no hypoxia compared to vehicle control and vinorelbine-treated tumors (Fig. 5c). No significant difference in microvessel density (*p* = 0.1072), lectin perfusion (*p* = 0.4096), or tumor hypoxia was observed between FGF401- and FGF401/vinorelbine-treated tumors, suggesting that vinorelbine does not antagonize the effects of FGF401 on blood vessel normalization. Taken together, these observations indicate that treatment with FGF401 alone or in combination with vinorelbine slows tumor growth, improves vascular function, and decreases tumor hypoxia.

**Fig. 6:**
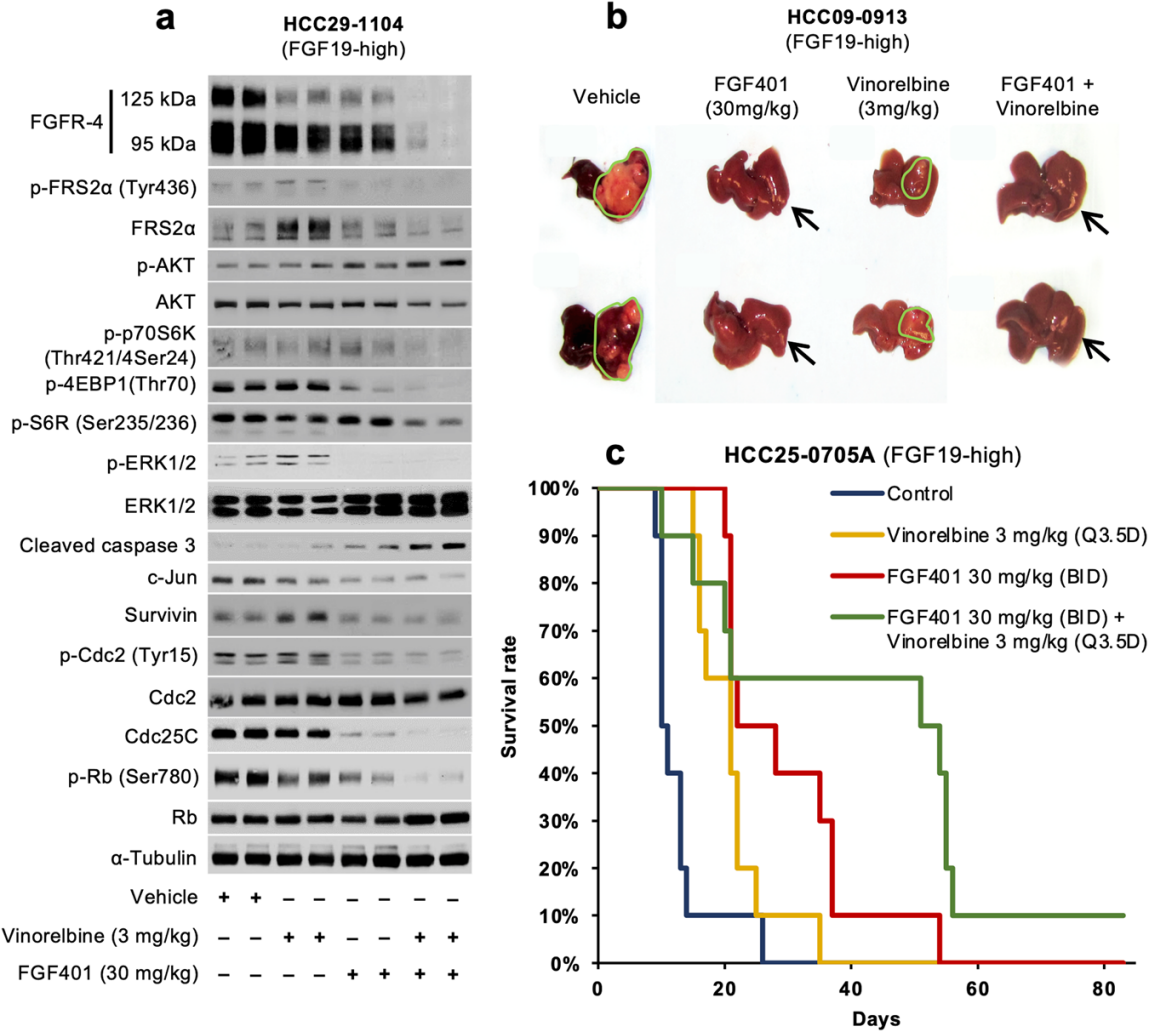
Effects of FGF401, vinorelbine and FGF401 plus vinorelbine on tumor growth, overall survival of mice and the FGFR-4 signaling pathway in high FGF19-expressing HCC tumors. HCC29-1104 tumors were subcutaneously implanted, and mice were treated for 4 days with vehicle, FGF401, vinorelbine, or their combination. Tumors were collected 2 h after the last dose of treatment, and tumor lysates were subjected to Western blot analysis (**a**). Mice bearing HCC25-0705A and HCC09-0913 orthotopic tumors were treated daily as described for 28 days. The sizes of the primary orthotopic tumors (**b**) and Kaplan–Meier survival analyses (**c**) are shown.

The FGF401/vinorelbine combination decreased the expression of FGFR-4 with a molecular weight of 125 kDa in HCC09-0913 tumors compared with FGF401 or vinorelbine alone, indicating reduced phosphorylation and glycosylation. This in turn further reduced the activation of p-FRS-2α, p-p70S6K Thr421/Ser424, p-4EBP1 Thr70, p-Erk1/2, pRb Ser780, Cdc25C and c-Jun and increased the levels of p-Akt Ser473 and cleaved caspase 3 (Fig. 6a and Supplementary Fig. 6). Vinorelbine treatment alone increased the levels of p-FRS-2α, survivin, and p-Cdc2; however, these effects were eliminated by the addition of FGF401.

Partial inhibition of tumor growth was observed following vinorelbine treatment relative to the vehicle control, while FGF401 potently inhibited tumor growth in orthotopic models (Fig. 6b). The combination of vinorelbine and FGF401 significantly improved the antitumor efficacy compared with FGF401 alone (Fig. 6b, *p* < 0.05). No significant differences in body weight loss or other clinical signs of toxicity were observed between treatment groups.

Kaplan–Meier survival analysis showed that 90% of mice bearing HCC25-0705A tumors in the vehicle, vinorelbine, and FGF401 groups were moribund on days 18, 24, and 38, respectively (Fig. 6c). The survival time was significantly longer in the FGF401/vinorelbine combination group, where 90% of the mice were moribund on day 58 (Fig. 6c, *p* < 0.001, log-rank test).

## Discussion

The diagnosis of HCC portends a dismal diagnosis, with a high incidence in Asia and the developing world. HCCs in the early stages are potentially curable, but most present in advanced stages, where chemotherapy and targeted agents are clinically ineffective^1,25^. Sorafenib^2,3^, cabozantinib^26^, and lenvatinib^4^ have been approved by the FDA for the treatment of patients with advanced HCC. Various targeted agents, including everolimus^27^, sunitinib^28^, and brivanib^29^, failed to reach their primary endpoint of improved OS, possibly due to studies conducted in unselected populations without any predictive biomarkers.

It has been proposed that the amplification of 11q13, which contains FGF19, is present in approximately 6 to 12% of HCC samples and leads to the activation of FGFR-4^16,30–32^. In addition, serum FGF19 levels in HCC patients are higher than those in healthy individuals^5,10^. In our cohort, overexpression of FGF19 occurred in 9 of 63 (14.3%) HCC models tested, of which only 1 of 9 high FGF19-expressing HCC models (HCC29-1104) had FGF19 gene amplification. The mechanism(s) responsible for the overexpression of FGF19 in 8 HCC models with no FGF19 gene amplification remains to be elucidated.

In the context of FGF19-dependent tumors, FGF19/FGFR-4 blockade with FGF401 resulted in tumor growth inhibition, reduction of cell proliferation, induction of apoptosis, and improved OS in both orthotopic and ectopic models. Consistent with previous reports^33,34^, we demonstrated that FGF401 exerts potent antitumor activity in all 9 high FGF19-expressing models. The HCC10-1212, HCC10-0112B, HCC26-1004, and HCC2-1318 models, which have a diploid FGF19 copy number, low levels of FGFR-4 protein, and high levels of FGF19 protein, are quite sensitive to FGF401, suggesting that FGF19 overexpression is a reliable marker for FGF401. Therefore, immunostaining for tumor FGF19 or measuring serum FGF19 could be a valuable and practical method for identifying patients with HCC who will likely respond to FGF401.

Mechanistically, FGF401 causes G1 cell cycle arrest and apoptosis probably by reducing Bcl-XL, with a concomitant increase in Bim, a potent inducer of apoptosis (Supplementary Fig. 5b). One of the antiapoptotic effects of the MEK/ERK pathway is mediated by the Erk1/2 phosphorylation of Bim (reviewed in ref. ^35^). FGF401 and FGF401/vinorelbine inhibit p-ERK1/2^36^, therefore leading to the dephosphorylation of Bim, as indicated by a shift in migration. Inhibition of the p70S6K pathways^37^ by FGF401 has also been observed.

We further found that the antitumor effects of FGF401 are the result of its dual mechanisms of action: acting on HCC cells and altering the tumor microenvironment. While blood vessels in vehicle-treated tumors are hyperdilated and nonproductive, the dense capillary-like network of vessels in FGF401-treated tumors is slim, regularly shaped and productive, as determined by lectin perfusion. These findings suggest that the reduction in intratumoral hypoxia is due to the ability of FGF401 to restore and increase oxygen supply to the tumor via blood vessel normalization. Coupled with the reduction in cell proliferation and elevation in apoptosis, this results in significant antitumor efficacy. In contrast, FGF401 was unable to induce normalization in the HCC17-0211 and HCC26-0808A models, which express FGFR2-4 but not FGF19. As a result, extensive hypoxia and necrosis occurred mainly at the center of the tumor.

The exact mechanisms underlying FGF401-induced blood vessel normalization remain to be elucidated. It is important to consider that FGF19/FGFR-4 signaling can alter the tumor microenvironment by modulating circulating endothelial cells via FGF19-dependent paracrine mechanisms and possibly soluble factors. Endothelial cells survive, proliferate, and reorganize to form capillary-like blood vessels in response to soluble factors that are produced by FGF401-treated tumor cells. As FGF401 treatment continues, more productive blood vessels form and restore the local oxygen concentration. Thus, a regulated balance of growth factors in tumor vascular networks can generate a hierarchy of well-organized and functional vessels. Previously, angiogenesis-based cancer treatment focused on blocking angiogenesis or pruning the vasculature to improve tumor perfusion and oxygenation^38–40^. However, our data suggest an alternative therapeutic approach based on promoting blood vessel “normalization”.

To our knowledge, this is the first study to report that blockade of the FGF19/FGFR-4 signaling pathway leads to an improvement in the tumor microenvironment through blood vessel normalization. In addition, FGF401 treatment results in elevated serum ALT, ALP, and AST levels and a significant decrease in serum creatinine levels (Supplementary Table 1), consistent with the safety profiles of FGF401 in human studies^20^. Thus, targeted therapies aimed at blocking the autocrine/paracrine FGF19/FGFR-4 loop might serve as effective treatments in a subset of HCC patients with FGF19/FGFR-4-driven tumors. In fact, several FGFR-4 inhibitors, such as FGF401, AZ709, H3B6527, and ASP5878^19,33,41^, are currently being tested in clinical trials. Recently, a phase I study of fisogatinib in patients with FGF19-positive HCC showed promising antitumor activity^18^.

We also demonstrated that FGF401 significantly sensitizes tumors to the antimitotic chemotherapy drug vinorelbine, presumably through normalizing blood vessels, allowing more efficient drug delivery. Importantly, vinorelbine did not alter FGF401-induced blood vessel normalization. Attenuating or antagonizing the vinorelbine-induced elevation in p-FRS-2α, p-Cdc2, and survivin and the total inhibition of CDC25C and c-Jun may contribute in part to the potent antitumor activity observed with FGF401/vinorelbine treatment. These observations are consistent with previous studies showing that vessel normalization reduces tumor invasion and dissemination and increases tumor responses to chemotherapeutic agents^42–44^. Experiments are ongoing to determine the potential of combining FGF401-induced vessel normalization with immune checkpoint inhibitors to improve immunotherapy^45–47^.

The HCC lines HCC13-0109, HCC26-0808A, and HCC17-0211, which express FGFR1-4 and Klotho β but not FGF19, are sensitive to infigratinib (pan-FGFR inhibitor) but not FGF401. This is presumably due to genetic alterations in other FGF signaling genes, such as FGFR-2 and −3^21^, but not those in the FGF19/FGFR-4 axis. Thus, FGF401 treatment is not expected to have the same toxicity profile as infigratinib but may be associated with an increased risk of cholestatic injury in patients who already have dysregulated bile acid homeostasis^48^. Whether the administration of FGF401 would lead to similar clinical symptoms as previously observed with the anti-FGF19 antibody remains to be determined^49^. Considering that FGF401 has a shorter plasma half-life than the antibody, the dosing regimen of FGF401 can be optimized to minimize toxicity.

In summary, using a panel of HCC PDX models, we showed that high FGF19 expression predicts sensitivity to FGF401. FGF401 induces tumor regression in high FGF19-expressing HCC models by modulating FGF19/FGFR-4 signaling, inhibiting proliferation, inducing apoptosis, and inhibiting hypoxia via blood vessel normalization, and this effect was further enhanced by the addition of vinorelbine. Preliminary data suggest that FGF401 has a manageable safety profile, warranting further investigations on the efficacy of FGF401 and FGF401/vinorelbine in clinical settings. To date, promising clinical activity has been observed in patients with advanced HCC in a phase 1 study of FGF401. Studies to further evaluate FGF401 as a single agent and in combination are ongoing^20^.

## Supplementary information

Supplementary Information

## References

[1] Tunissiolli NM (2017). Hepatocellular carcinoma: a comprehensive review of biomarkers, clinical aspects, and therapy. Asian Pac. J. Cancer Prev..

[2] Cheng A-L (2009). Efficacy and safety of sorafenib in patients in the Asia-Pacific region with advanced hepatocellular carcinoma: a phase III randomised, double-blind, placebo-controlled trial. Lancet Oncol..

[3] Llovet JM (2008). Sorafenib in advanced hepatocellular carcinoma. N. Engl. J. Med..

[4] Kudo M (2018). Lenvatinib versus sorafenib in first-line treatment of patients with unresectable hepatocellular carcinoma: a randomised phase 3 non-inferiority trial. Lancet (London, England).

[5] Miura S (2012). Fibroblast growth factor 19 expression correlates with tumor progression and poorer prognosis of hepatocellular carcinoma. BMC Cancer.

[6] Guagnano V (2012). FGFR genetic alterations predict for sensitivity to NVP-BGJ398, a selective pan-FGFR inhibitor. Cancer Discov..

[7] Katoh M, Nakagama H (2014). FGF receptors: cancer biology and therapeutics. Med. Res. Rev..

[8] Goetz R (2007). Molecular insights into the klotho-dependent, endocrine mode of action of fibroblast growth factor 19 subfamily members. Mol. Cell. Biol..

[9] Desnoyers LR (2008). Targeting FGF19 inhibits tumor growth in colon cancer xenograft and FGF19 transgenic hepatocellular carcinoma models. Oncogene.

[10] Hyeon J, Ahn S, Lee JJ, Song DH, Park C-K (2013). Expression of fibroblast growth factor 19 is associated with recurrence and poor prognosis of hepatocellular carcinoma. Dig. Dis. Sci..

[11] Zhou M (2014). Separating tumorigenicity from bile acid regulatory activity for endocrine hormone FGF19. Cancer Res..

[12] French DM (2012). Targeting FGFR4 inhibits hepatocellular carcinoma in preclinical mouse models. PLoS ONE.

[13] Ho HK (2009). Fibroblast growth factor receptor 4 regulates proliferation, anti-apoptosis and alpha-fetoprotein secretion during hepatocellular carcinoma progression and represents a potential target for therapeutic intervention. J. Hepatol..

[14] Chen Z (2013). FGFR4 and TGF-beta1 expression in hepatocellular carcinoma: correlation with clinicopathological features and prognosis. Int. J. Med. Sci..

[15] Gauglhofer C (2014). Fibroblast growth factor receptor 4: a putative key driver for the aggressive phenotype of hepatocellular carcinoma. Carcinogenesis.

[16] Sawey ET (2011). Identification of a therapeutic strategy targeting amplified FGF19 in liver cancer by oncogenomic screening. Cancer Cell.

[17] Lin BC, Desnoyers LR (2012). FGF19 and cancer. Adv. Exp. Med. Biol..

[18] Kim RD (2019). First-in-human phase i study of fisogatinib (BLU-554) validates aberrant FGF19 signaling as a driver event in hepatocellular carcinoma. Cancer Discov..

[19] Weiss A (2017). Abstract 2103: NVP-FGF401: cellular and in vivo profile of a novel highly potent and selective FGFR4 inhibitor for the treatment of FGF19/FGFR4/KLB+ tumors. Cancer Res..

[20] Chan SL (2017). Abstract CT106: Ph I/II study of FGF401 in adult pts with HCC or solid tumors characterized by FGFR4/KLB expression. Cancer Res..

[21] Huynh H (2019). Infigratinib mediates vascular normalization, impairs metastasis, and improves chemotherapy in hepatocellular carcinoma. Hepatology.

[22] Huynh H, Soo KC, Chow PKH, Panasci L, Tran E (2006). Xenografts of human hepatocellular carcinoma: a useful model for testing drugs. Clin. Cancer Res..

[23] Huynh H (2010). AZD6244 (ARRY-142886) enhances the antitumor activity of rapamycin in mouse models of human hepatocellular carcinoma. Cancer.

[24] Gonzalez-Cid M, Larripa I, Slavutsky I (1997). Vinorelbine: cell cycle kinetics and differential sensitivity of human lymphocyte subpopulations. Toxicol. Lett..

[25] Tovoli F (2018). Systemic treatments for hepatocellular carcinoma: challenges and future perspectives. Hepatic Oncol..

[26] Abou-Alfa GK (2018). Cabozantinib (C) versus placebo (P) in patients (pts) with advanced hepatocellular carcinoma (HCC) who have received prior sorafenib: Results from the randomized phase III CELESTIAL trial. J. Clin. Oncol..

[27] Zhu AX (2014). Effect of everolimus on survival in advanced hepatocellular carcinoma after failure of sorafenib: the EVOLVE-1 randomized clinical trial. JAMA.

[28] Cheng A-L (2013). Sunitinib versus sorafenib in advanced hepatocellular cancer: results of a randomized phase III trial. J. Clin. Oncol..

[29] Johnson PJ (2013). Brivanib versus sorafenib as first-line therapy in patients with unresectable, advanced hepatocellular carcinoma: results from the randomized phase III BRISK-FL study. J. Clin. Oncol..

[30] Roessler S (2012). Integrative genomic identification of genes on 8p associated with hepatocellular carcinoma progression and patient survival. Gastroenterology.

[31] Chiang DY (2008). Focal gains of VEGFA and molecular classification of hepatocellular carcinoma. Cancer Res..

[32] Huang J, Manning BD (2008). The TSC1-TSC2 complex: a molecular switchboard controlling cell growth. Biochem. J..

[33] Futami T (2017). ASP5878, a Novel Inhibitor of FGFR1, 2, 3, and 4, Inhibits the Growth of FGF19-Expressing Hepatocellular Carcinoma. Mol. Cancer Ther..

[34] Weiss A (2019). FGF401, A First-In-Class Highly Selective and Potent FGFR4 Inhibitor for the Treatment of FGF19-Driven Hepatocellular Cancer. Mol. Cancer Ther..

[35] Steelman LS (2011). Roles of the Raf/MEK/ERK and PI3K/PTEN/Akt/mTOR pathways in controlling growth and sensitivity to therapy-implications for cancer and aging. Aging (Albany NY)..

[36] Wiesenauer CA, Yip-Schneider MT, Wang Y, Schmidt CM (2004). Multiple anticancer effects of blocking MEK-ERK signaling in hepatocellular carcinoma. J. Am. Coll. Surg..

[37] Huynh H (2009). RAD001 (everolimus) inhibits tumour growth in xenograft models of human hepatocellular carcinoma. J. Cell. Mol. Med..

[38] Lee CG (2000). Anti-Vascular endothelial growth factor treatment augments tumor radiation response under normoxic or hypoxic conditions. Cancer Res..

[39] Jain RK (2002). Tumor angiogenesis and accessibility: role of vascular endothelial growth factor. Semin. Oncol..

[40] Jain RK (2005). Antiangiogenic therapy for cancer: current and emerging concepts. Oncol. (Williston Park)..

[41] Selvaraj A (2017). Abstract 3126: H3B6527, a selective and potent FGFR4 inhibitor for FGF19-driven hepatocellular carcinoma. Cancer Res..

[42] Jain RK (2005). Normalization of tumor vasculature: an emerging concept in antiangiogenic therapy. Science.

[43] Park J-S (2016). Normalization of tumor vessels by Tie2 activation and Ang2 inhibition enhances drug delivery and produces a favorable tumor microenvironment. Cancer Cell.

[44] Mpekris F, Baish JW, Stylianopoulos T, Jain RK (2017). Role of vascular normalization in benefit from metronomic chemotherapy. Proc. Natl Acad. Sci. USA..

[45] Fukumura D, Kloepper J, Amoozgar Z, Duda DG, Jain RK (2018). Enhancing cancer immunotherapy using antiangiogenics: opportunities and challenges. Nat. Rev. Clin. Oncol..

[46] Khan KA, Kerbel RS (2018). Improving immunotherapy outcomes with anti-angiogenic treatments and vice versa. Nat. Rev. Clin. Oncol..

[47] Tian L (2017). Mutual regulation of tumour vessel normalization and immunostimulatory reprogramming. Nature.

[48] Mellor HR (2014). Targeted inhibition of the FGF19-FGFR4 pathway in hepatocellular carcinoma; translational safety considerations. Liver Int..

[49] Pai R (2012). Antibody-mediated inhibition of fibroblast growth factor 19 results in increased bile acids synthesis and ileal malabsorption of bile acids in cynomolgus monkeys. Toxicol. Sci..

