# Supplementary material for: FGF401 and vinorelbine synergistically mediate antitumor activity and vascular normalization in FGF19-dependent hepatocellular carcinoma
Source: Exp Mol Med. 2020 Nov 25;52(11):1857–68. doi: 10.1038/s12276-020-00524-4 (PMC8080677; doi:10.1038/s12276-020-00524-4)
Supplement: Supplementary file 1 — Supplementary Information [file 12276_2020_524_MOESM1_ESM.pdf]

Supplementary Figure Legends

Supplementary Figure 1. Expression of FGF19, FGFR-4 and Klotho-β across a panel of 63 human HCC PDX models. Tumor samples were collected from 63 HCC PDX models and were homogenized in buffer containing 50 mM Tris-HCl pH 7.4, 150 mM NaCl, 0.5% NP-40, 1 mM EDTA, and 25 mM NaF, supplemented with proteinase inhibitors and 10 mM Na3VO4. Eighty micrograms of proteins per sample were analyzed by Western blot. Blots were incubated with antibodies against FGF19, FGFR-4, Klotho β, and α-tubulin. Representative blots are shown (**a**). DNA and RNA from 15 HCC PDX and 2 normal livers were extracted. DNA samples were genotyped using Affymetrix SNP array 6.0 and RNA samples were subjected to RNAseq to determine mRNA levels (**b**).

Supplementary Figure 2. Duration of FGFR-4 pathway inhibition and the effects of FGF401 on cell proliferation, apoptosis and angiogenesis in HCC PDX models. HCC09-0913-bearing mice were dosed with 60 mg/kg of FGF401 and tumor collected at the indicated hours. Lysates of 2 independent tumors were subjected to Western blot analysis and representative blots and quantitative analysis are shown (**a**). Indicated tumors were subcutaneously implanted and mice were treated with vehicle or 30 mg/kg FGF401 (BID) for 14-16 days. Tumors were collected 2 hours after the last treatments and stained for p-Histone H3 Ser10, cleaved PARP and CD31. Representative photographs of tumor sections are shown (**b**).

Supplementary Figure 3. Effect of FGF401 on blood vessel normalization in high FGF19-expressing HCC26-1004. Tissue section from HCC26-1004 tumor treated with vehicle, 15 mg/kg infigratinib, or 30 mg/kg FGF401 were stained for blood vessel (CD31 and lectin). Representative image of tumor sections are shown.

Supplementary Figure 4. Antitumor activity of FGF401 in high and low FGF19-expressing HCC PDX models. Tumors were implanted subcutaneously and mice were treated with vehicle or 30 mg/kg FGF401 (BID) (n=10 mice per treatment group). Tumor volumes were calculated and mean volumes ± SEs at the indicated time points are shown. $p<0.05$ is considered to be significant (Student' t-test).

Supplementary Figure 5. Effect of FGF401 on FGFR signaling pathways. Human umbilical vascular endothelial cells (HUVEC) were treated with vehicle or 2 nM FGF401 for 24 hours. Protein were collected and changes in the levels of proteins were determined by Western blotting. HCC21-0208 cell lysate was used as positive control (**a**). Mice were subcutaneously implanted with HCC30-0805B and HCC29-1104 tumor and treated with vehicle or 30 mg/kg FGF401. Tumors were collected and lysates were subjected to Western blot analysis. Representative blots are shown (**b**) . FGF401 caused a reduction in FGF19 and subsequent decrease in p-Erk1/2 and p-p90RSK Thr359/Ser363, indicative of reduced

cell growth. There was also a decrease in Bcl-xL and concomitant increase in Bim and cleaved capsase 3, suggesting an increase in apoptosis. Inhibition of the p70S6K pathways by FGF401 is also observed.

Supplementary Figure 6. Effects of FGF401, vinorelbine and FGF401 plus vinorelbine on the FGFR-4 signaling pathway in high FGF19-expressing HCC tumors. HCC09-0913 and HCC13-0212 tumors were subcutaneously implanted and mice bearing tumors of approximately 700-800 mm^3 were treated for 4 days with vehicle, FGF401, vinorelbine, or the combination of both. Tumors were collected 2h after the last dose of treatment and tumor lysates were subjected to Western blot analysis.

Supplementary Materials and Methods

Reagents. Antibodies against AKT (#9272), p70S6K (#9202), Survivin (#2803), Rb (#9313), Cdk2 (#2546), Cyclin B1 (#4138), CDC25C (#4688), cleaved caspase 3 (#9661), cleaved PARP (#5625), Cdc2 (#9112), α-tubulin (#2144) and phosphorylation-specific antibodies against Rb Ser807/811 (#9308), AKT Ser473 (#9271), FRS-2α Tyr439 (#3861), p70S6K Thr421/424 (#9204), S6R Ser235/236 (#2211), 4EBP1 Thr70 (#9455), Histone 3 Ser10 (#9701), Cdc2 Tyr15 (#9111), and ERK1/2 Thr202/Tyr204 (#4370) were obtained from Cell Signaling Technology (Beverly, MA, USA). The antibodies against ERK1/2 (sc-94), FRS2-α (sc-17841), and p-Cdk2 Thr14/Tyr15 (sc-28435-R) were from Santa Cruz Biotechnology Inc. (Santa Cruz, CA, USA). Anti-mouse CD31 (#2502) antibody was from BioLegend (San Diego, CA, USA). FGF401 was from Novartis (Basel, Switzerland). Infigratinib were purchased from Selleck Chemicals, Houston, TX, USA. Navelbine (Vinorelbine) 10 mg/ml was obtained from Pierre Fabre Medicament (Boulogne Cedex, France). Sorafenib was from Bayer (South East Asia) Pte Ltd, Singapore.

Real-time quantitative RT-PCR analysis

The expressions of FGF19 and FGFR-4 were examined by reverse transcription polymerase chain reaction (RT-PCR) with the following primers:

**Target gene (human)**	**Forward (5’-3’)**	**Reverse (5’-3’)**
FGF19	CAGCTGTACAAGAACAGAGGCTTTC	AAATGGGTCCATGCTGTCGGTCTCC
FGFR-4	CATCCGCTGGCTTAAGGATGGAC	ATCACGAGACTCCAGTGCTGATG
GAPDH	TCTCCTCTGACTTCAACAGCGACAC	TGTTGCTGTAGCCAAATTCGTTGTC

All qPCR reactions were performed using the SYBR Green PCR Core Reagents kit (Perkin-Elmer Applied Biosystems, Foster City, CA, USA) under the following conditions: 1 cycle at 95°C for 10 min, 50 cycles at 95°C for 10 sec, 60°C for 5 sec, 72°C for 10 sec, and 80°C for 1 sec. Real-time detection of the SYBR Green emission intensity was conducted with a LightCycler® (Roche, Mannheim, Germany). Equal amount of cDNA sample derived from 40 ng total RNA, was used for each PCR reaction. The mRNA in each sample was then quantified with reference to the standard curve constructed with each use of the LightCycler®. Quantitative RT-PCR was performed at least three times per sample. To standardize the amount of RNA, expression of GAPDH mRNA in each sample was quantified and the amounts of expressed FGF19 and FGFR-4 mRNA were divided by GAPDH mRNA levels.

Cell culture. Fresh HCC09-0913, HCC13-0109 and HCC13-0212 cells were isolated from PDX tumors and maintained as monolayer cultures in high glucose Dulbecco's Modified Eagle's Medium (DMEM) supplemented with 10% fetal bovine serum (FBS) and 1% penicillin-streptomycin at 37°C with 5% CO2.

Flow cytometry analysis. HCC09-0913, HCC13-0109 and HCC13-0212 cells were plated at the density of 5 x 10^5 and then treated with either 0.1% DMSO or 0.5, 1 and 1.5 µM FGF401 for 24 hours. Cells were trypsinized and fixed in 70% ethanol at 4°C for 24 hours and stained with propidium iodide. The fluorescence intensities of the stained cells were measured using a FACSCalibur flow cytometer (BD, San Jose, CA, USA). Data were analyzed using BD CellQuest Pro software. For every measurement, 10,000 events were collected, and gating was set to exclude cell doublets. The DNA contents of certain phases are shown as percentages compared to the total DNA content within the gate.

Xenograft models. This study received ethics board approval at the SingHealth and National Cancer Centre Singapore. All animals received humane care according to the criteria outlined in the "Guide for the Care and Use of Laboratory Animals" prepared by the National Academy of Sciences and published by the National Institutes of Health (NIH publication 86-23 revised 1985) (49).

HCC PDX xenograft lines were used to establish tumors in male C.B-17 SCID mice aged 9-10 weeks, weighing 23-25 g (InVivos Pte. Ltd., Singapore) (Huynh et al., 2019, 2006). Mice were provided with sterilized food and water ad libitum and housed in negative pressure isolators set at 23°C and 43% humidity, with 12-h light/dark cycles.

Sixty-three previously established xenograft lines (Huynh et al., 2006) were used to screen for the expression of FGFR-4, FGF19 and Klotho-β by Western blot analysis. Xenograft lines with high and low FGF19 were selected to establish tumors in male SCID mice (InVivos Pte Ltd., Singapore).

The efficacy of FGF401 and FGF401/Vinorelbine combination was determined by the T/C ratio, where T and C are the median weights of drug-treated and vehicle-treated tumors at the end of treatment, respectively. T/C ratios less than 0.42 were considered active as determined according to the criteria of the Drug Evaluation Branch of the Division of Cancer Treatment, National Cancer Institute.

Vessel perfusion study. Each mouse bearing tumor xenografts (vehicle- or drug-treated) was intravenously injected with 100 µg of biotinylated Lycopersicon esculentum (tomato) lectin (VectorLabs #B-1175) prepared in 100 µl of 0.9% NaCl. The tumors were harvested 10 min after lectin perfusion, fixed in 10% formalin and processed for paraffin embedding

before obtaining 5μm sections. Peroxidase activity and nonspecific staining were blocked, and the sections were incubated for 1 hour at room temperature with streptavidin peroxidase. To visualize productive microvessels, immunohistochemistry was performed using the streptavidin-biotin peroxidase complex method, according to the manufacturer's instructions (Lab Vision Corporation, Fremont, CA, USA). For the quantifications of the mean microvessel density in sections, 10 random 0.159-mm^2 fields at a magnification of 100X were captured for each tumor.

To determine the extent of hypoxia in tumor tissues, mice bearing tumors (vehicle- and drug-treated) were intraperitoneally injected with 60 mg/kg pimonidazole hydrochloride 1 hour before tumor harvest. Hypoxic regions of tumors were identified by staining the sections with Hypoxyprobe plus Kit HP2 according to the manufacturer's instructions (Hypoxyprobe Inc, Burlington, MA, USA).

Western blot analysis. To determine the changes in protein expressions, tumors from vehicle- or drug-treated mice were homogenized in buffer containing 50 mM Tris-HCl pH 7.4, 150 mM NaCl, 0.5% NP-40, 1 mM EDTA, and 25 mM NaF, supplemented with protease inhibitors and 10 mM Na3VO4. Eighty micrograms of protein per sample was resolved by SDS-PAGE and transferred to a nitrocellulose membrane (Huynh, 2010; Huynh et al., 2019, 2006). Blots were incubated with primary antibodies followed by horseradish peroxidase-conjugated secondary antibodies. Blots were visualized with a chemiluminescent detection system (Amersham, Pharmacia Biotech). Total band densities were subsequently quantified, normalized to α-tubulin and expressed as the fold change relative to the control (vehicle-treated sample). A value greater (or less) than 1 indicated that the expression level of the protein of interest was greater (or less) than that of the control group.

Statistical analysis. Differences in the levels of protein bands, the tumor weight at sacrifice, p-Histone 3 Ser10 index, mean microvessel density, and the number of cleaved PARP-positive cells were compared. Student's t-test was used for the comparison between two groups. One-way analysis of variance followed by the Tukey-Kramer method post-hoc test was used when comparing more than two groups. For survival analysis, the log-rank test was used. $p<0.05$ indicates a statistically significant difference. Graphs were generated using Prism 8 v. 8.2.1.

Supplementary Table 1. Blood composition analysis from mice treated with vehicle, 30 mg/kg FGF401 twice a day, 3 mg/kg vinorelbine every 3.5 days or the combination of both.

	**Vehicle**	**FGF401**	**Vinorelbine**	**FGF401 + Vinorelbine**
**BUN (mg/dL)**	14	15	14.5	12.5
**CRE (mg/dL)**	0.45	0.2	0.35	0.2
**ALT (U/L)**	38.5	332	49.5	190.5
**ALP (U/L)**	54	66.5	58	73
**AST (U/L)**	193	468.5	249	279
**TBIL (mg/dL)**	0.3	0.3	0.3	0.3
**GLU (mg/dL)**	162.5	172	136.5	160
**CA (mg/dL)**	10.05	9.8	9.85	10
**TP (g/dL)**	5.15	5.15	5.05	5.2
**ALB (g/dL)**	4	4.1	3.7	4.05
**5GLOB (g/dL)**	1.15	1.05	1.45	1.25
**Na^+ (mmol/L)**	146.5	147.5	149	146
**K^+ (mmol/L)**	8.35	8.35	8.25	8.25
**Cl^- (mmol/L)**	105.5	107.5	107.5	106.5
**tCO_2 (mmol/L)**	25.5	25.5	22.5	26
**WBC (10^{-9}/L)**	2.88	4.41	2.03	1.50
**LYM (10^{-9}/L)**	0.69	1.99	0.4	0.295
**MON (10^{-9}/L)**	0.185	0.31	0.165	0.16
**NEU (10^{-9}/L)**	2.01	2.11	1.575	1.035
**LYM (%)**	23.8	45.1	20.9	19.85
**MON (%)**	6.1	7.1	9.3	11.05
**NEU (%)**	70.1	47.8	69.8	69.1
**RBC (10^{-12}/L)**	10.975	11.40	10.08	8.78
**HGB (g/dl)**	16.75	18.3	15	12.45
**HCT (%)**	51.54	54.07	46.97	40.36
**MCV (fl)**	47	47	46.5	46
**MCH (pg)**	15.25	16	14.9	14.15
**MCHC (g/dl)**	32.5	33.8	31.95	30.75
**RDWc (%)**	16.9	17.2	17.8	17.65
**RDWs (fl)**	31.2	32	32.8	32.4
**PLT (10^{-9}/L)**	226	210	282.5	218.5
**MPV (fl)**	6.2	5.9	6.75	6.45
**PCT (%)**	0.14	0.12	0.195	0.104
**PDWc (%)**	30.95	27.9	34.1	32.9
**PDWs (fl)**	8.05	6.4	10.2	9.4

Supplementary Table 2. Mean tumor weight ± SEM from mice treated with vehicle or 30 mg/kg FGF401 twice daily. Statistical analyses were performed using Student's t-test and significance was established at $p<0.05$.

Xenograft Line	Levels of FGF19	Treatments	Mean tumor weight (g) ± SEM	*p*-value (Vehicle vs. FGF401)
HCC01-0207	High	Vehicle	1.3242 ± 0.1275	$p = 1.2335 \times 10^{-7}$
		FGF401 30 mg/kg BID	0.1714 ± 0.025	
HCC09-0913	High	Vehicle	1.5097 ± 0.1079	$p = 1.2131 \times 10^{-10}$
		FGF401 30 mg/kg BID	0.0894 ± 0.0105	
HCC29-1104	High	Vehicle	1.4863 ± 0.1028	$p = 1.4624 \times 10^{-9}$
		FGF401 30 mg/kg BID	0.2946 ± 0.0264	
HCC26-0808B	High	Vehicle	2.1969 ± 0.1203	$p = 9.3814 \times 10^{-10}$
		FGF401 30 mg/kg BID	0.520 9± 0.0510	
HCC25-0705A	High	Vehicle	1.4443 ± 0.1137	$p = 2.4195 \times 10^{-9}$
		FGF401 30 mg/kg BID	0.1611 ± 0.0290	
HCC26-1104	High	Vehicle	2.3026 ± 0.2677	$p = 1.9661 \times 10^{-5}$
		FGF401 30 mg/kg BID	0.4948 ± 0.1038	
HCC2-1318	High	Vehicle	2.0998 ± 0.1806	$p = 1.3688 \times 10^{-7}$
		FGF401 30 mg/kg BID	0.3203 ± 0.0449	
HCC10-1212	High	Vehicle	1.2844 ±0.1335	$p = 0.0002710$
		FGF401 30 mg/kg BID	0.472± 0.082	
HCC10-0112B	High	Vehicle	0.7281 ± 0.0854	$p = 2.4931 \times 10^{-7}$
		FGF401 30 mg/kg BID	0.0451 ± 0.0043	
HCC13-0212	High	Vehicle	2.3347 ± 0.1808	$p = 2.6582 \times 10^{-6}$
		FGF401 30 mg/kg BID	1.0128 ± 0.0773	
HCC30-0805B	High	Vehicle	1.8557 ± 0.1544	$p = 2.4424 \times 10^{-8}$
		FGF401 30 mg/kg BID	0.1332 ± 0.0105	
HCC05-0614	Low	Vehicle	0.6951 ± 0.0930	$p = 0.009621$
		FGF401 30 mg/kg BID	0.3886 ± 0.0504	
HCC01-0214	Low	Vehicle	1.6304 ± 0.1923	$p = 0.004352$
		FGF401 30 mg/kg BID	0.9405 ± 0.884	
HCC26-0808A	Low	Vehicle	1.2528 ± 0.1344	$p = 0.1507$
		FGF401 30 mg/kg BID	0.9874 ± 0.1012	
HCC06-0606	Low	Vehicle	2.6143 ± 0.2934	$p = 0.1283$
		FGF401 30 mg/kg BID	2.0774 ± 0.1371	
HCC21-0208	Low	Vehicle	1.8415 ± 0.1726	$p = 3.1595 \times 10^{-5}$
		FGF401 30 mg/kg BID	0.7299 ± 0.1048	
HCC25-0707A LQN-R	High	Vehicle	2.1229 ± 0.2398	$p = 0.2950$
		FGF401 30 mg/kg BID	1.6759 ± 0.3348	

Supplementary Table 3. Mean tumor burden ± SEM between mice treated with vehicle, 30 mg/kg FGF401, 3 mg/kg vinorelbine, or the combination of both. Statistical analyses were performed using Student's t-test and significance was established at *p*<0.05.

**Xenograft line**	**Mean tumor burden (g) ± SEM**				***p*-value**
	**Vehicle**	**FGF401 (30 mg/kg)**	**Vinorelbine (3 mg/kg)**	**FGF401 + Vinorelbine**	
HCC26-0808B (FGF19-high)	1.921 ± 0.2424	0.3635 ± 0.0414	0.8622 ± 0.0767	0.1614 ± 0.0290	Veh vs. FGF401 ***p*<0.001**; Veh vs. Vino ***p*<0.001**; Veh vs. FG401/Vino ***p*<0.0001**; FGF401 vs. Vino ***p*<0.001**; FGF401 vs. FGF401/Vino ***p*<0.001**; Vino vs. FGF401/Vino ***p*<0.0001**
HCC29-1104 (FGF19-high)	1.8520 ± 0.1164	0.3718 ±0.0313	0.9082 ± 0.1622	0.1001 ± 0.0142	Veh vs. FGF401 ***p*<0.0001**; Veh vs. Vino ***p*<0.001**; Veh vs. FG401/Vino ***p*<0.0001**; FGF401 vs. Vino ***p*<0.001**; FGF401 vs. FGF401/Vino ***p*<0.0001**; Vino vs. FGF401/Vino ***p*<0.001**
HCC25-0707A (FGF19-high)	1.3722 ± 0.2452	0.2425 ± 0.0368	0.6703 ± 0.086	0.0468 ± 0.0083	Veh vs. FGF401 ***p*<0.001**; Veh vs. Vino ***p*<0.05**; Veh vs. FG401/Vino ***p*<0.001**; FGF401 vs. Vino ***p*<0.001**; FGF401 vs. FGF401/Vino ***p*<0.001**; Vino vs. FGF401/Vino ***p*<0.001**
HCC09-0913 (FGF19-high)	1.9802 ± 0.2042	0.1378 ± 0.0140	1.2888 ± 0.1049	0.1115 ± 0.0073	Veh vs. FGF401 ***p*<0.0001**; Veh vs. Vino ***p*<0.01**; Veh vs. FG401/Vino ***p*<0.0001**; FGF401 vs. Vino ***p*<0.0001**; FGF401 vs. FGF401/Vino ***p*=0.1186**; Vino vs. FGF401/Vino ***p*<0.0001**
HCC01-0207 (FGF19-high)	1.8214 ± 0.2645	0.5818 ± 0.128	1.6940 ± 0.1852	0.2776 ± 0.0911	Veh vs. FGF401 ***p*<0.001**; Veh vs. Vino ***p*=0.4345**; Veh vs. FG401/Vino ***p*<0.001**; FGF401 vs. Vino ***p*<0.001**; FGF401 vs. FGF401/Vino ***p*<0.05**; Vino vs. FGF401/Vino ***p*<0.001**
HCC26-1004 (FGF19-high)	1.4655 ± 0.1018	0.4511 ± 0.1203	1.4410 ± 0.1128	0.1845 ± 0.043	Veh vs. FGF401 ***p*<0.01**; Veh vs. Vino ***p*=0.2632**; Veh vs. FG401/Vino ***p*<0.001**; FGF401 vs. Vino ***p*<0.01**; FGF401 vs. FGF401/Vino ***p*<0.01**; Vino vs. FGF401/Vino ***p*<0.001**
HCC2-1318 (FGF19-high)	1.8639 ± 0.1944	0.1033 ± 0.0078	1.2745 ± 0.1484	0.0509 ± 0.0044	Veh vs. FGF401 ***p*<0.0001**; Veh vs. Vino ***p*<0.05**; Veh vs. FG401/Vino ***p*<0.0001**; FGF401 vs. Vino ***p*<0.001**; FGF401 vs. FGF401/Vino ***p*<0.001**; Vino vs. FGF401/Vino ***p*<0.0001**
HCC10-1212 (FGF19-high)	1.1422 ± 0.267	0.1242 ±0.0164	0.727 ± 0.201	0.0724 ± 0.0092	Veh vs. FGF401 ***p*<0.001**; Veh vs. Vino ***p*=0.234**; Veh vs. FG401/Vino ***p*<0.001**; FGF401 vs. Vino ***p*<0.01**; FGF401 vs. FGF401/Vino ***p*<0.05**; Vino vs. FGF401/Vino ***p*<0.001**
HCC10-0112B (FGF19-high)	0.7281 ± 0.0854	0.0851 ±0.0043	0.5233 ± 0.0625	0.0366 ± 0.0059	Veh vs. FGF401 ***p*<0.0001**; Veh vs. Vino ***p*=0.06878**; Veh vs. FG401/Vino ***p*<0.0001**; FGF401 vs. Vino ***p*<0.0001**; FGF401 vs. FGF401/Vino ***p*<0.05**; Vino vs. FGF401/Vino ***p*<0.0001**
HCC13-0212 (FGF19-high)	1.9304 ± 0.2564	0.6822 ± 0.1632	0.4250 ± 0.072	0.1040 ± 0.0236	Veh vs. FGF401 ***p*<0.01**; Veh vs. Vino ***p*<0.001**; Veh vs. FG401/Vino ***p*<0.0001**; FGF401 vs Vino ***p*=0.1714**; FGF401 vs FGF401/Vino ***p*<0.01**; Vino vs FGF401/Vino ***p*<0.001**
HCC01-0214 (FGF19-low)	1.6304 ± 0.1923	0.9405 ± 0.0884	1.3892 ± 0.1173	0.6729 ± 0.0545	Veh vs. FGF401 ***p*<0.01**; Veh vs. Vino ***p*<0.05**; Veh vs. FG401/Vino ***p*<0.001**; FGF401 vs. Vino ***p*<0.01**; FGF401 vs. FGF401/Vino ***p*<0.05**; Vino vs. FGF401/Vino ***p*<0.001**

a

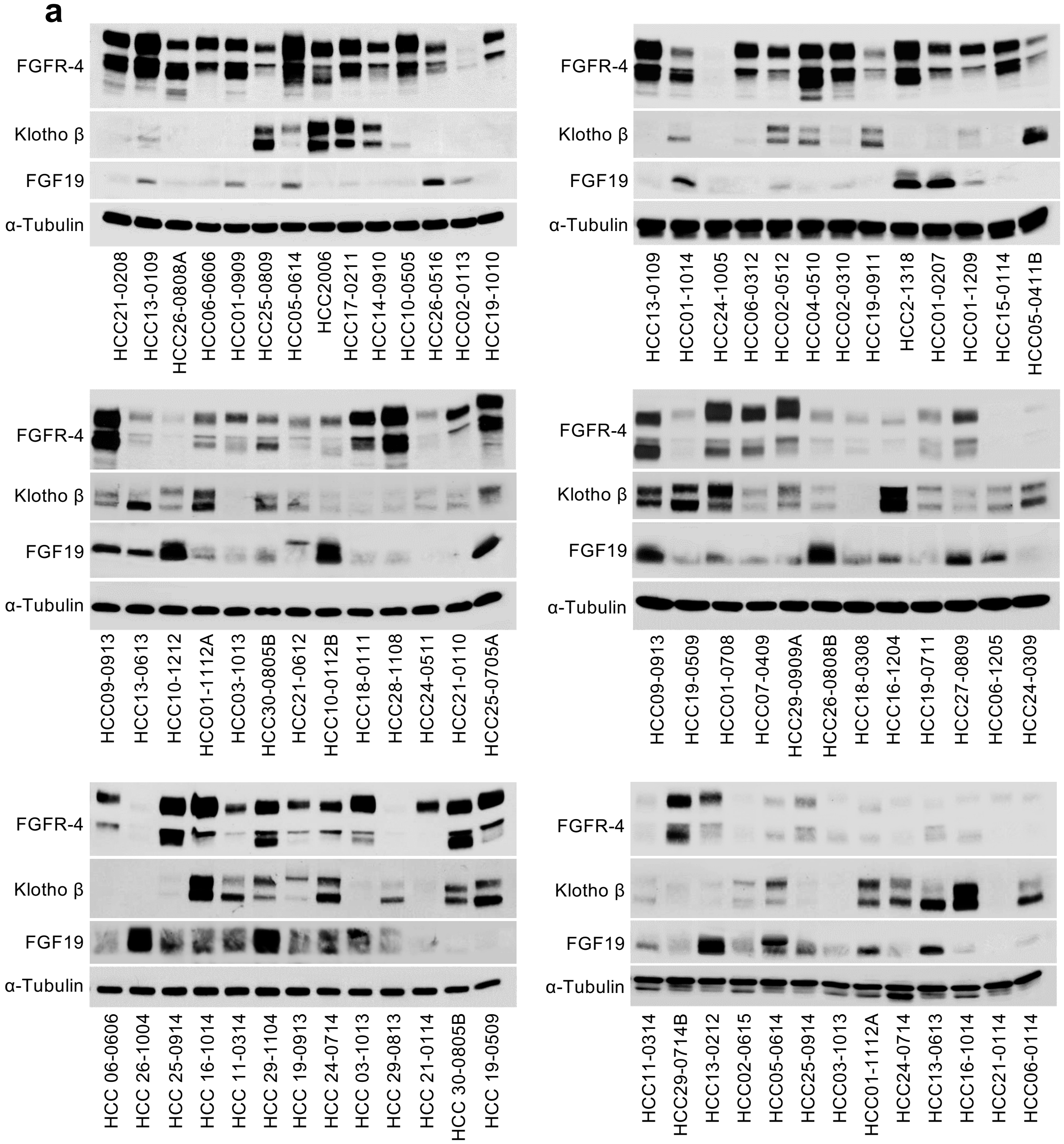

b

Gene	FGFR-4 copy	FGFR-4 mRNA	FGF19 mRNA
HCC01-0207	3	88.9	49.8
HCC09-0913	2	49.6	18.39
HCC25-0705A	4	189.9	37.74
HCC2-1318	2	48.87	16.36
HCC29-1104	2	64.54	197.46
HCC26-1004	2	70.5	13.61
HCC26-0808B	3	195.57	15.26
HCC10-1212	2	89.81	38.52
HCC10-0112B	2	58.40	18.19
HCC13-0212	2	90.68	14.33
HCC01-0909	2	172.62	0.98
HCC26-0808A	2	57.15	0.04
HCC06-0606	4	18.15	0.21
HCC13-0109	3	239.93	0.16
HCC17-0211	2	52.64	0
Normal liver 1973	2	20.71	0
Normal liver 328	2	20.50	0

Supplementary Figure 1

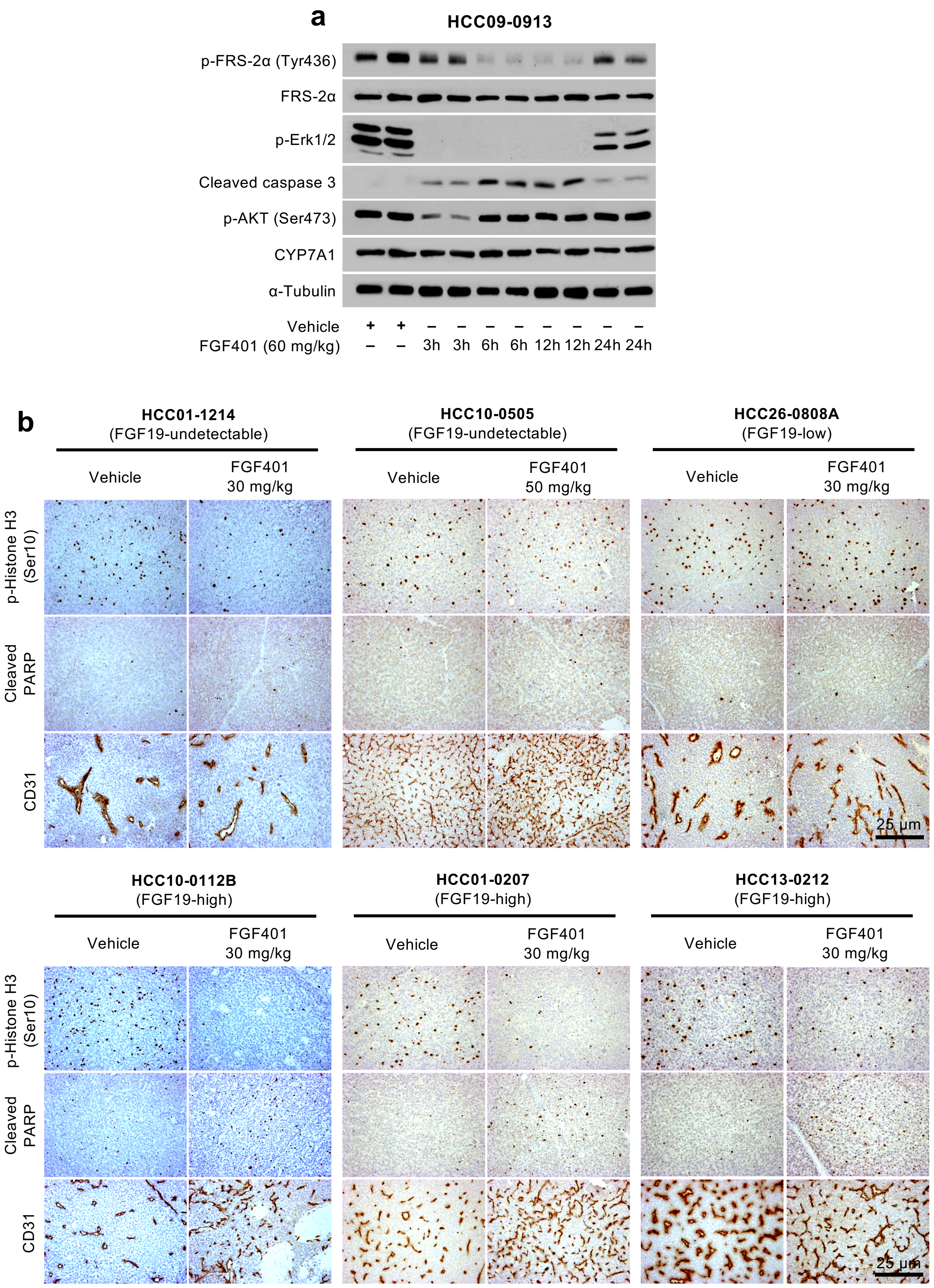

Supplementary Figure 2

HCC26-1004
(FGF19-high)

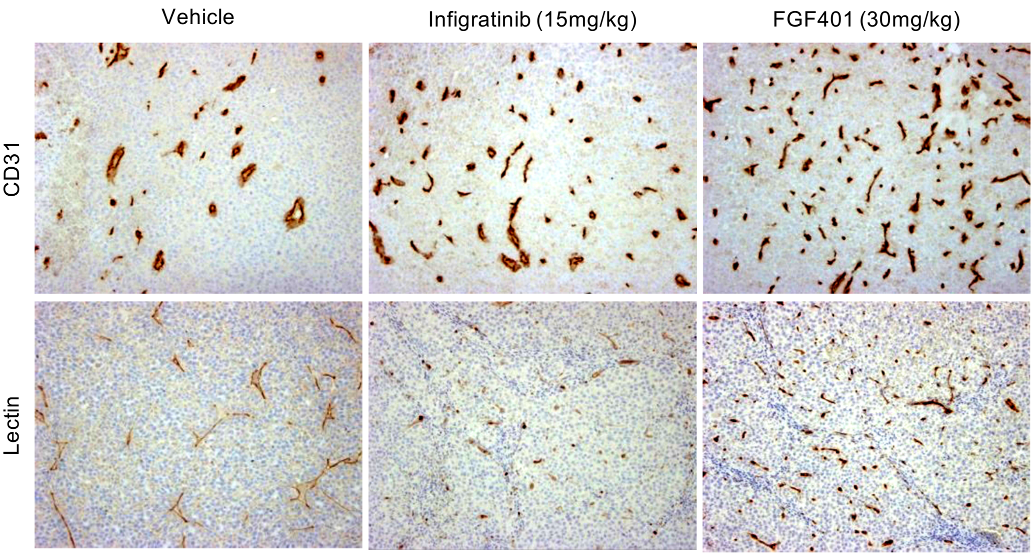

Supplementary Figure 3

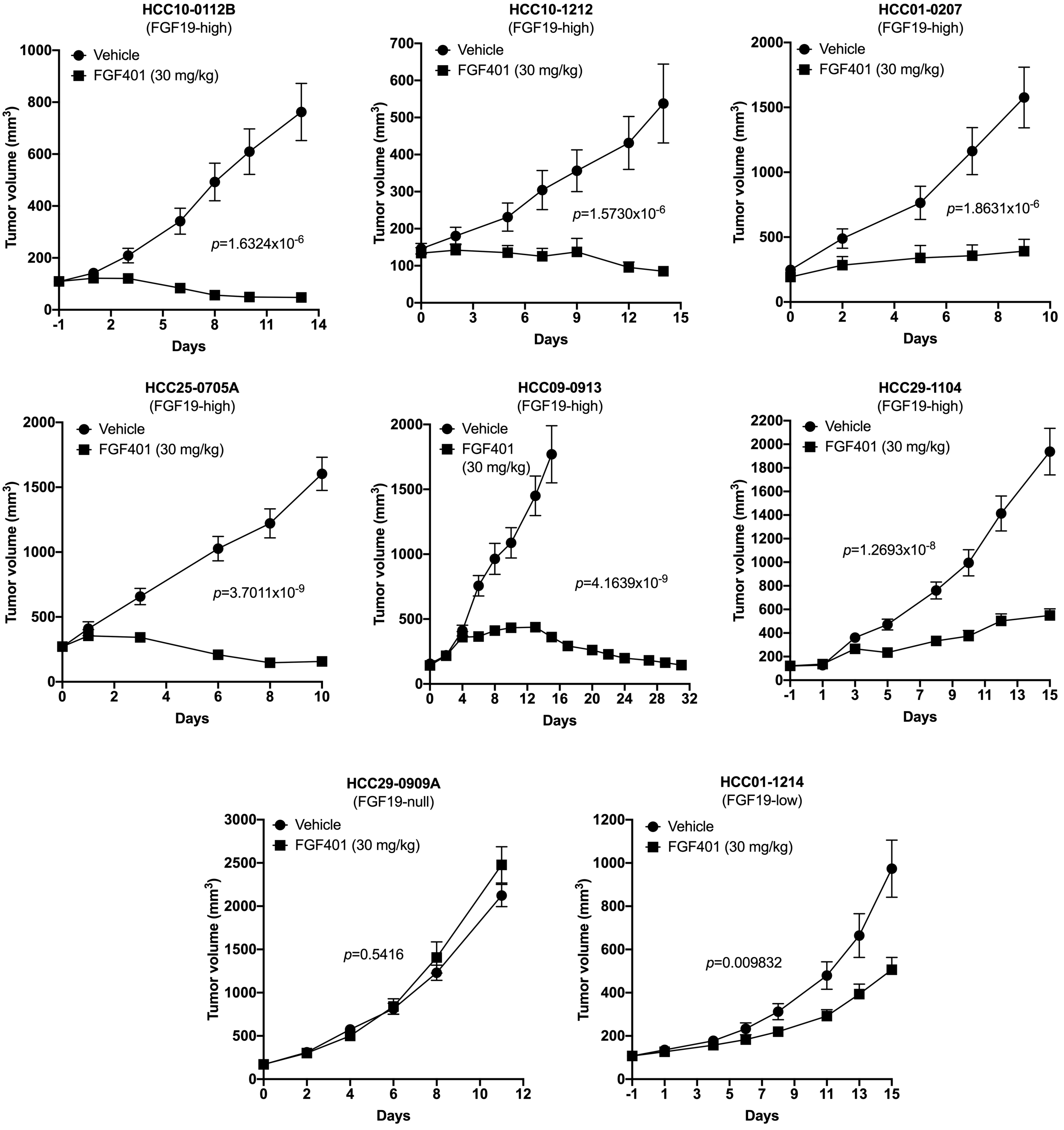

Supplementary Figure 4

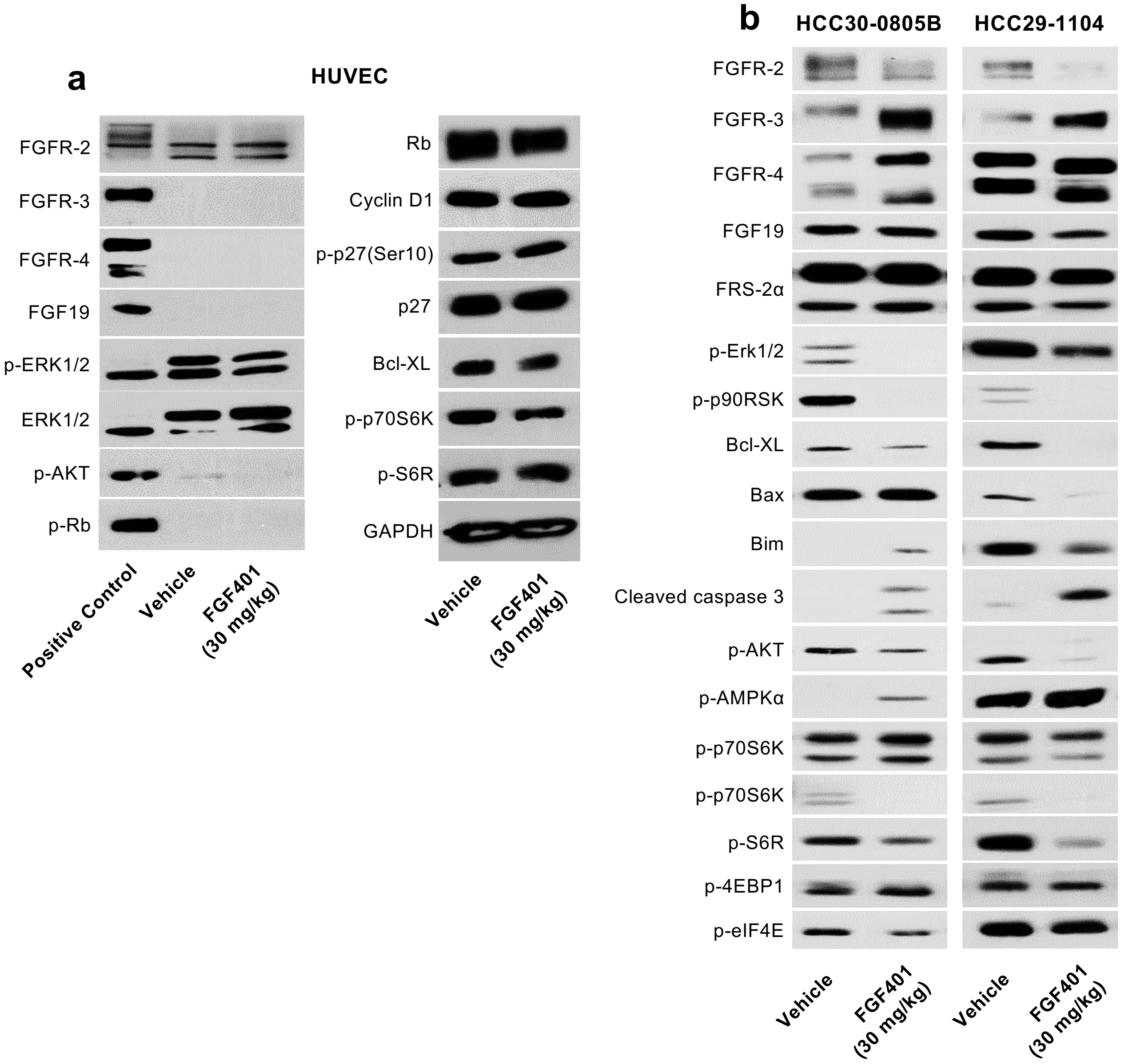

Supplementary Figure 5

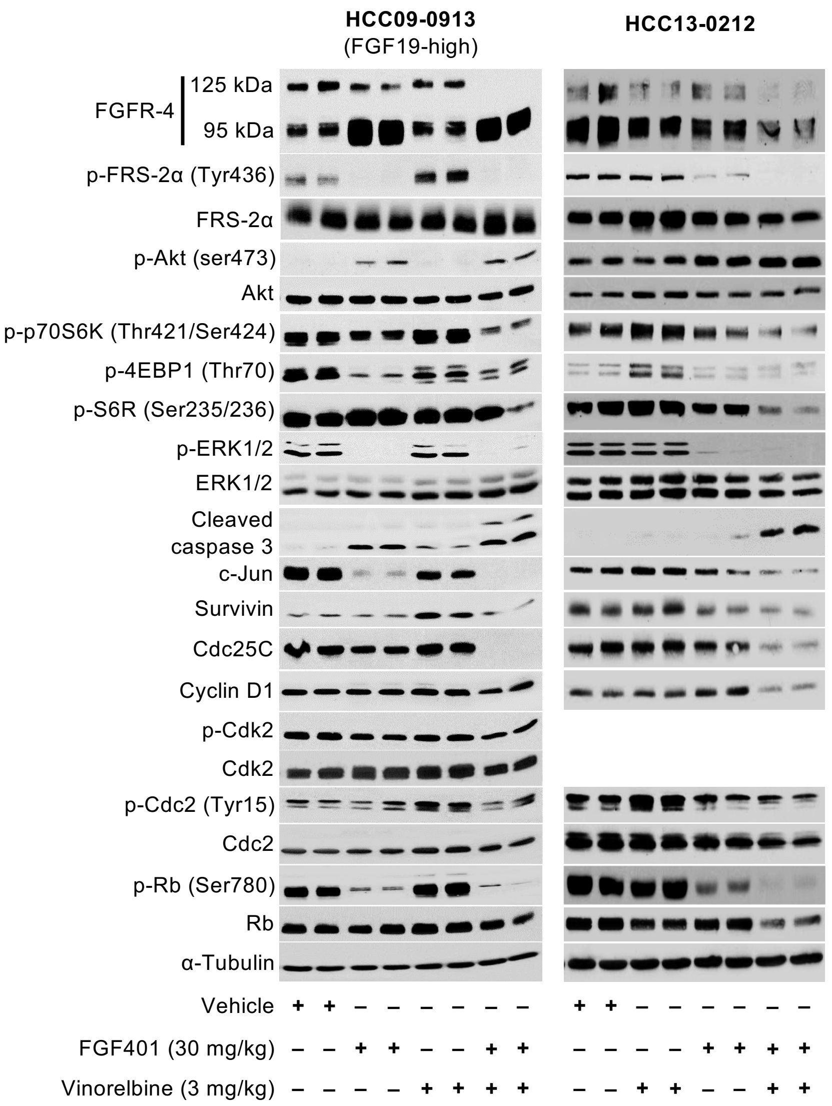

Supplementary Figure 6
